# Supplementary material for: Hydrate-melt electrolyte design for aqueous aluminium-bromine batteries with enhanced energy-power merits
Source: Nat Commun. 2025 Jul 9;16:6329. doi: 10.1038/s41467-025-61740-y (PMC12241333; doi:10.1038/s41467-025-61740-y)
Supplement: Supplementary file 1 — Supplementary Information [file 41467_2025_61740_MOESM1_ESM.pdf]

Hydrate-Melt Electrolyte Design for Aqueous Aluminium-Bromine Batteries with Enhanced Energy-Power Merits

Xingyuan Chu[1‡], Jingwei Du[1‡], Jiaxu Zhang[1], Xiaodong Li[1, 2], Xiaohui Liu[1], Yongkang Wang[3], Johannes Hunger[3], Ahiud Morag[1], Jinxin Liu[2], Quanquan Guo[1, 2], Dongqi Li[1], Yu Han[3], Mischa Bonn[3], Xinliang Feng[1, 2, *], Minghao Yu[1, 2, *]

[1] Center for Advancing Electronics Dresden (cfaed) & Faculty of Chemistry and Food Chemistry, Technische Universität Dresden, 01062, Dresden, Germany.

[2] Max Planck Institute of Microstructure Physics, 06120, Halle (Saale), Germany.

[3] Department of Molecular Spectroscopy, Max Planck Institute for Polymer Research, 55128, Mainz, Germany.

‡These authors contribute equally

*Corresponding author: xinliang.feng@tu-dresden.de; minghao.yu@tu-dresden.de

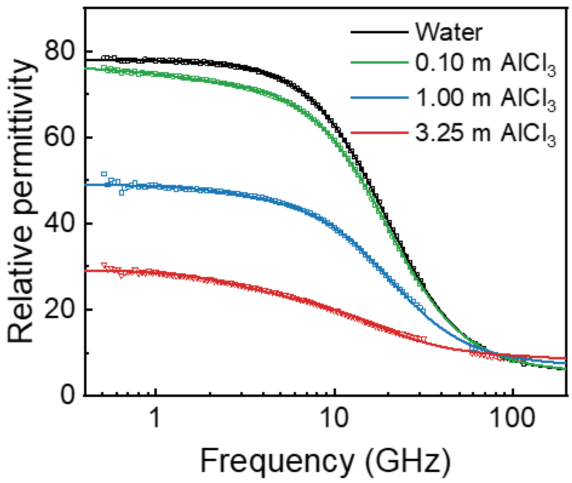

Supplementary Fig. 1 Dielectric permittivity spectra of aqueous $AlCl_3$ solutions at different concentrations. Symbols correspond to experimental data and solid lines show the fitting curves. Source data are provided as a Source Data file.

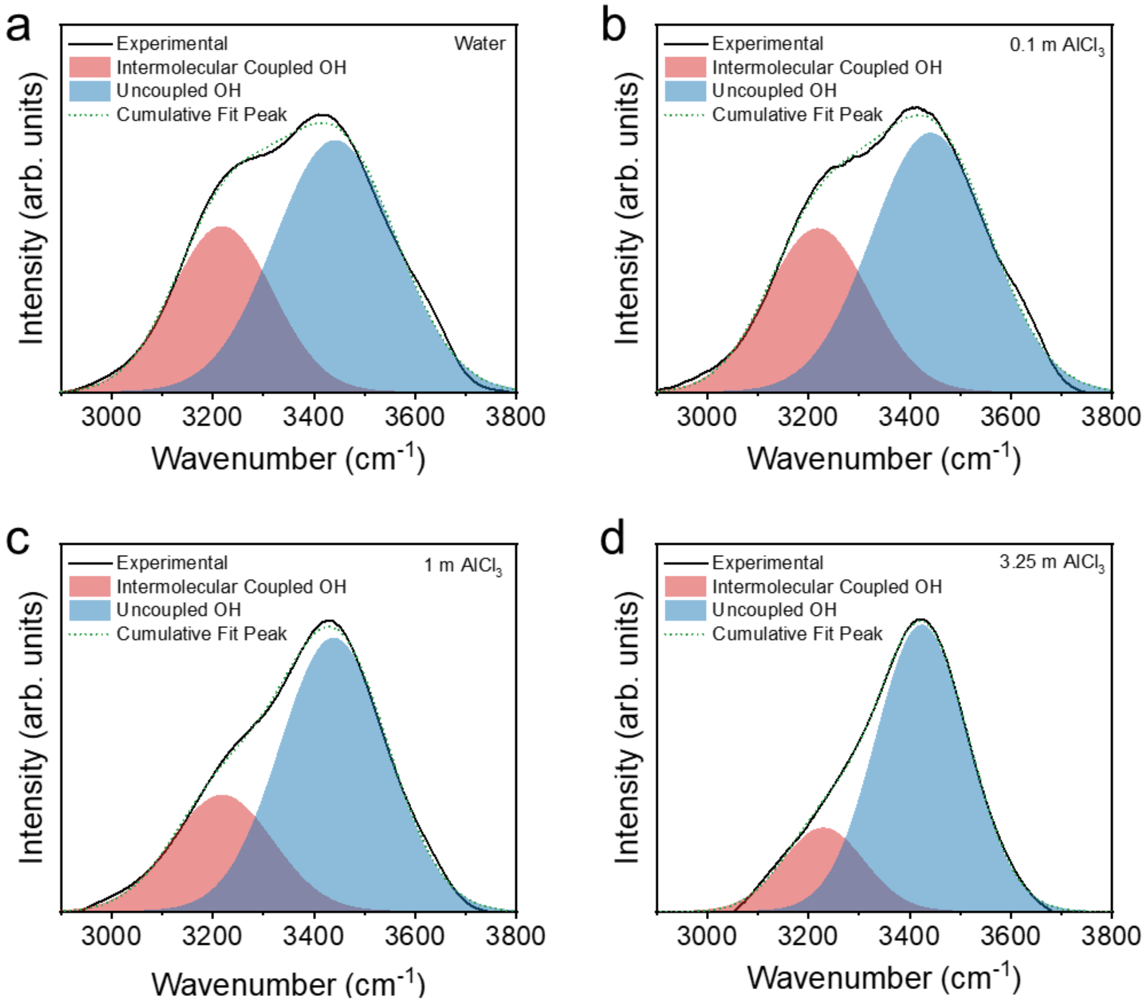

Supplementary Fig. 2 Raman spectra for **a** water, **b** 0.1 m $AlCl_3$, **c** 1 m $AlCl_3$ and **d** 3.25 m $AlCl_3$. Source data are provided as a Source Data file.

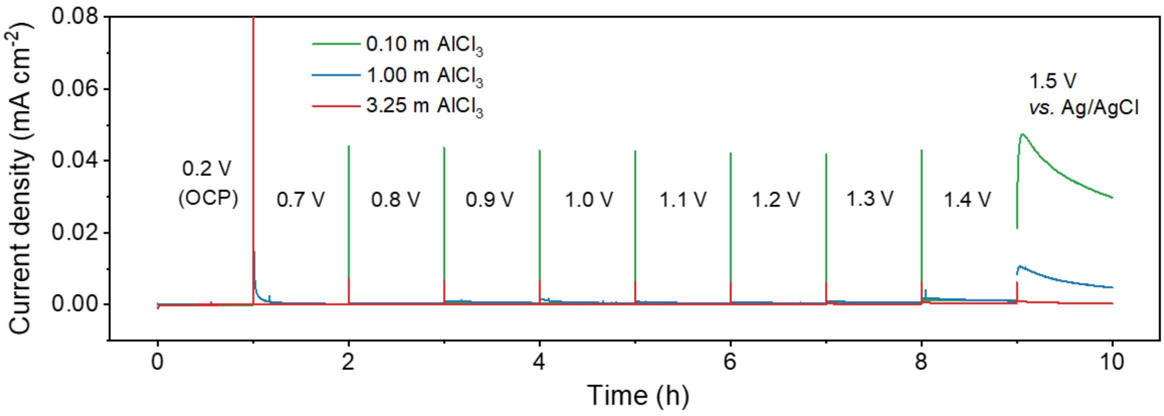

Supplementary Fig. 3 Potential floating test of the Ti electrode in varying $AlCl_3$ aqueous electrolytes. Source data are provided as a Source Data file.

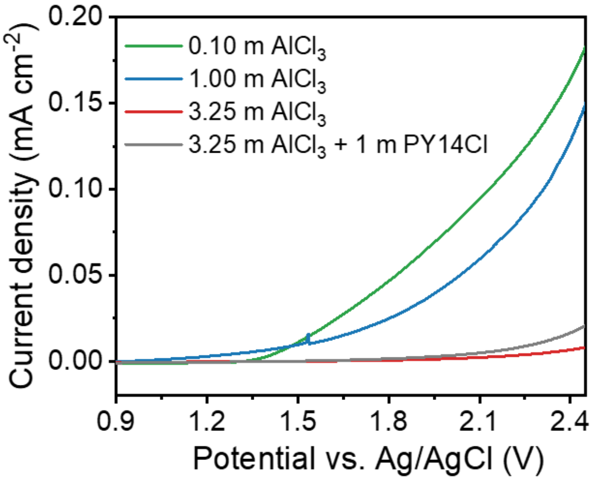

Supplementary Fig. 4 Linear scan voltametric curves of the Ti electrode in varying $AlCl_3$-based aqueous electrolytes. Source data are provided as a Source Data file.

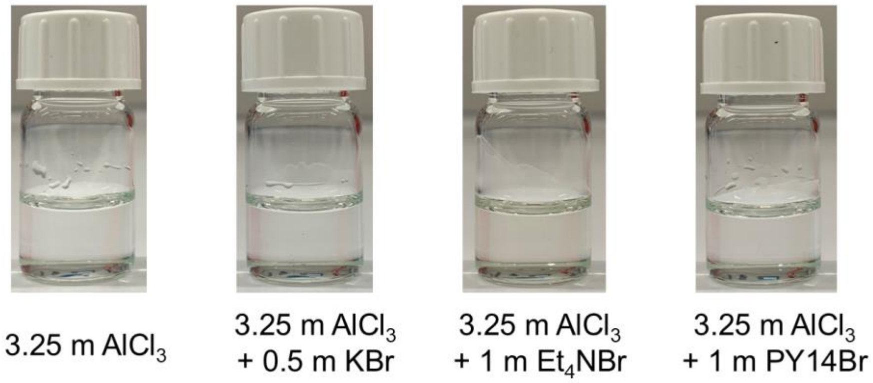

Supplementary Fig. 5 Digital photos of our electrolytes after three months of storage at room temperature.

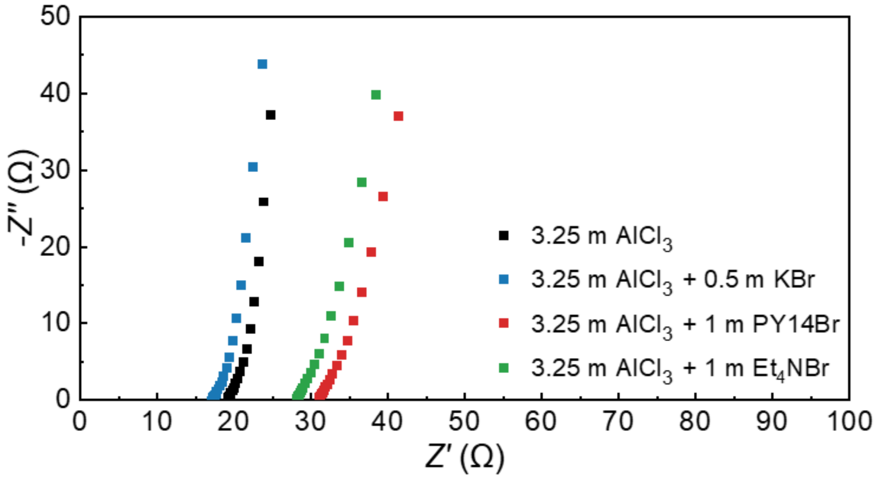

Supplementary Fig. 6 Electrochemical impedance spectroscopy analysis for 3.25 m $AlCl_3$, 3.25 m $AlCl_3$ + 0.5 m KBr, 3.25 m $AlCl_3$ + 1 m PY14Br, and 3.25 m $AlCl_3$ + 1 m Et_4NBr. The intercept along the real axis intrinsic shows the resistance of the electrolyte (R). The results are used for the calculation of electrolyte ionic conductivity. Source data are provided as a Source Data file.

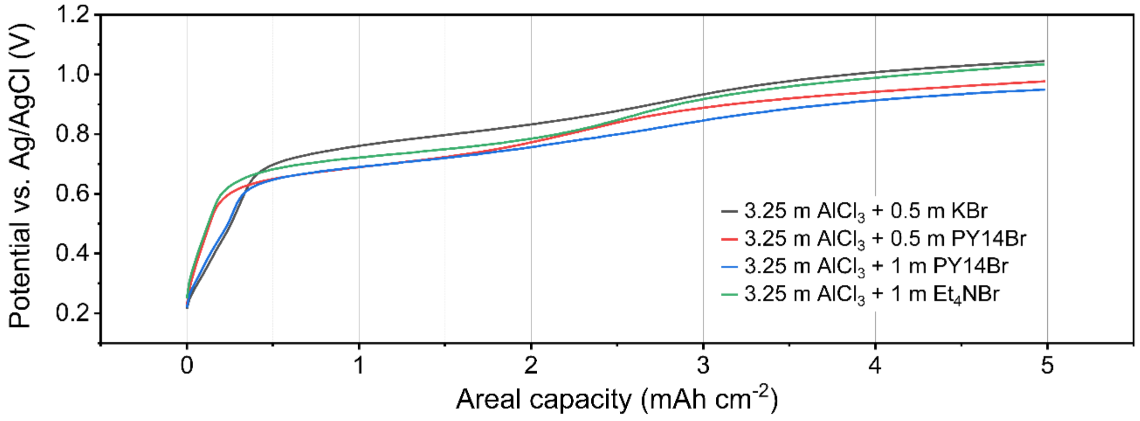

Supplementary Fig. 7 Galvanostatic charge curves of the AC electrode in different electrolytes at 5 mA cm^{-2} with 5 mAh cm^{-2} as the charge cut-off. Source data are provided as a Source Data file.

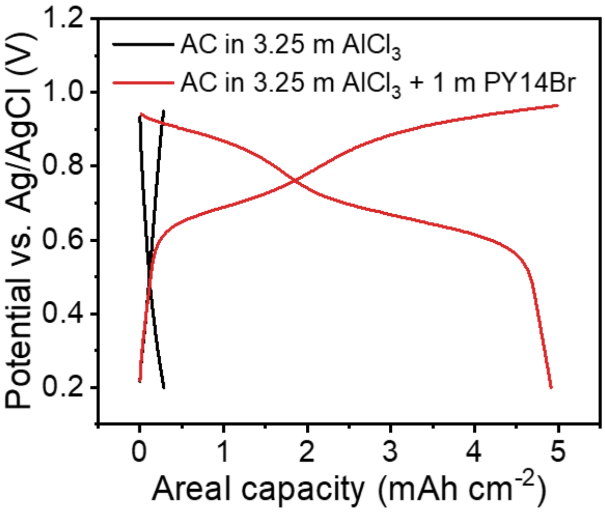

Supplementary Fig. 8 GCD profiles of the AC electrodes at 5 mA cm^{-2} in 3.25 m $AlCl_3$ and 3.25 m $AlCl_3$ + 1 m PY14Br. The capacitive charge storage of activated carbon accounts for only 0.28 mAh cm^{-2}, far below the total capacity of the AC electrode in 3.25 m $AlCl_3$ + 1 m PY14Br (4.97 mAh cm^{-2}). This result clearly confirms that the predominant capacity arises from the $Br^-/Br^0/Br^+$ conversion. Source data are provided as a Source Data file.

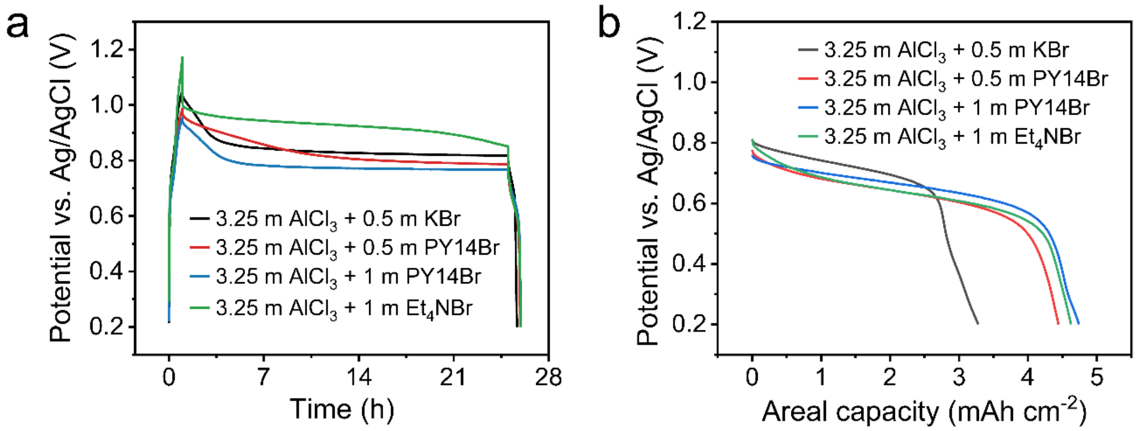

Supplementary Fig. 9 a Self-discharge test of the AC electrode in different electrolytes. The test was conducted by first charging the AC electrode at 5 mA cm^{-2} for 5 mAh cm^{-2}, then keeping open-circuit standing for 24 hours, and finally discharging the electrode at 5 mA cm^{-2} to 0.2 V *vs.* Ag/AgCl. **b** The final discharge curve of the AC electrode in the self-discharge test. Source data are provided as a Source Data file.

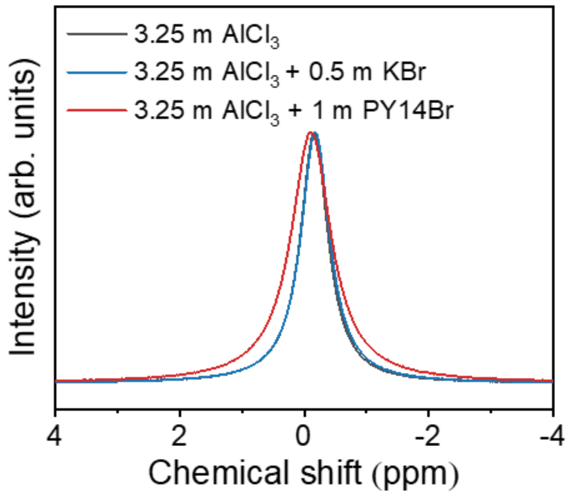

Supplementary Fig. 10 ^{27}Al NMR spectra of 3.25 m $AlCl_3$, 3.25 m $AlCl_3$ + 0.5 m KBr, and 3.25 m $AlCl_3$ + 1 m PY14Br. Source data are provided as a Source Data file.

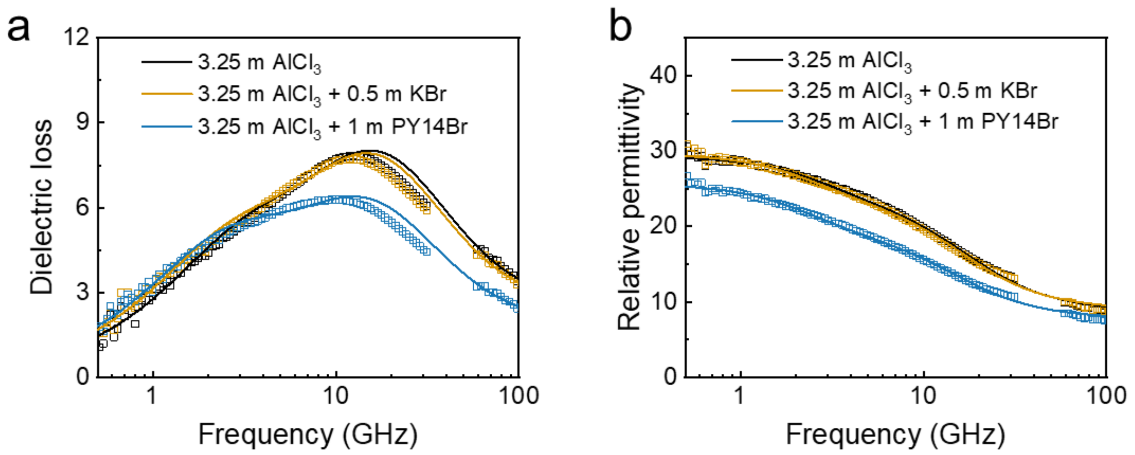

Supplementary Fig. 11 a Dielectric loss spectra and **b** relative permittivity of 3.25 m $AlCl_3$, 3.25 m $AlCl_3$ + 0.5 m KBr, and 3.25 m $AlCl_3$ + 1 m PY14Br. The contribution due to the dc conductivity to the loss spectra has been subtracted for visual clarity. Symbols correspond to experimental data and solid lines show the fitting curves. Source data are provided as a Source Data file.

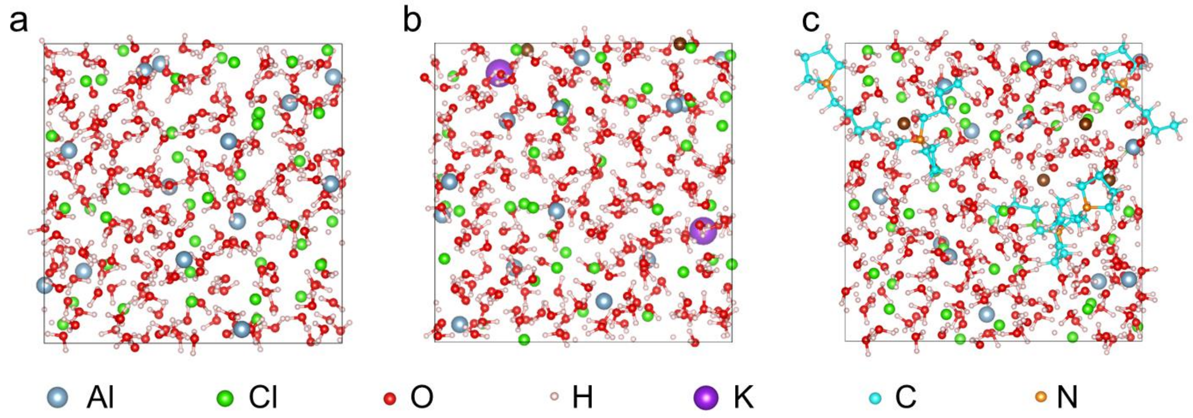

Supplementary Fig. 12 Snapshots from the AIMD simulations of **a** 3.25 m $AlCl_3$, **b** 3.25 m $AlCl_3$ + 0.5 m KBr, and **c** 3.25 m $AlCl_3$ + 1 m PY14Br. Blue spheres: Al, green spheres: Cl, red spheres: O, white spheres: H, brown spheres: Br, purple spheres: K, cyan spheres: C, orange spheres: N. The atomic coordinates are provided in Supplementary Data 1 (3.25 m $AlCl_3$), Supplementary Data 2 (3.25 m $AlCl_3$ + 0.5 m KBr) and Supplementary Data 3 (3.25 m $AlCl_3$ + 1 m PY14Br).

These snapshots serve as the foundation for extracting further information, such as simulated wide-angle X-ray scattering (WAXS) spectra (**Supplementary Fig. 13**), radial distribution function (RDF) data (**Fig. 2e-g** and **Supplementary Fig. 14-16**) and typical ion solvation configurations (**Supplementary Fig. 18**).

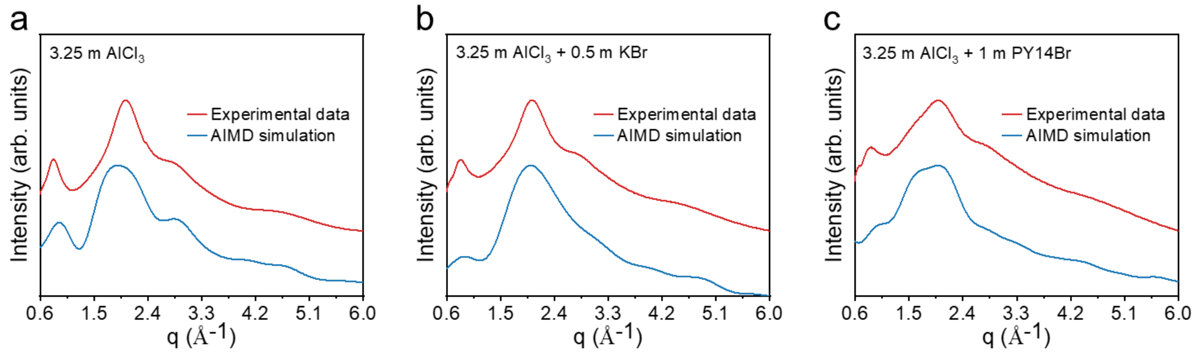

Supplementary Fig. 13 Experimental and AIMD-simulated WAXS spectra of **a** 3.25 m $AlCl_3$, **b** 3.25 m $AlCl_3$ + 0.5 m KBr, and **c** 3.25 m $AlCl_3$ + 1 m PY14Br. Source data are provided as a Source Data file.

The simulated spectra align well with the experimental spectra, demonstrating nearly identical spectral features. This strong agreement validates the reliability and accuracy of the AIMD simulation results.

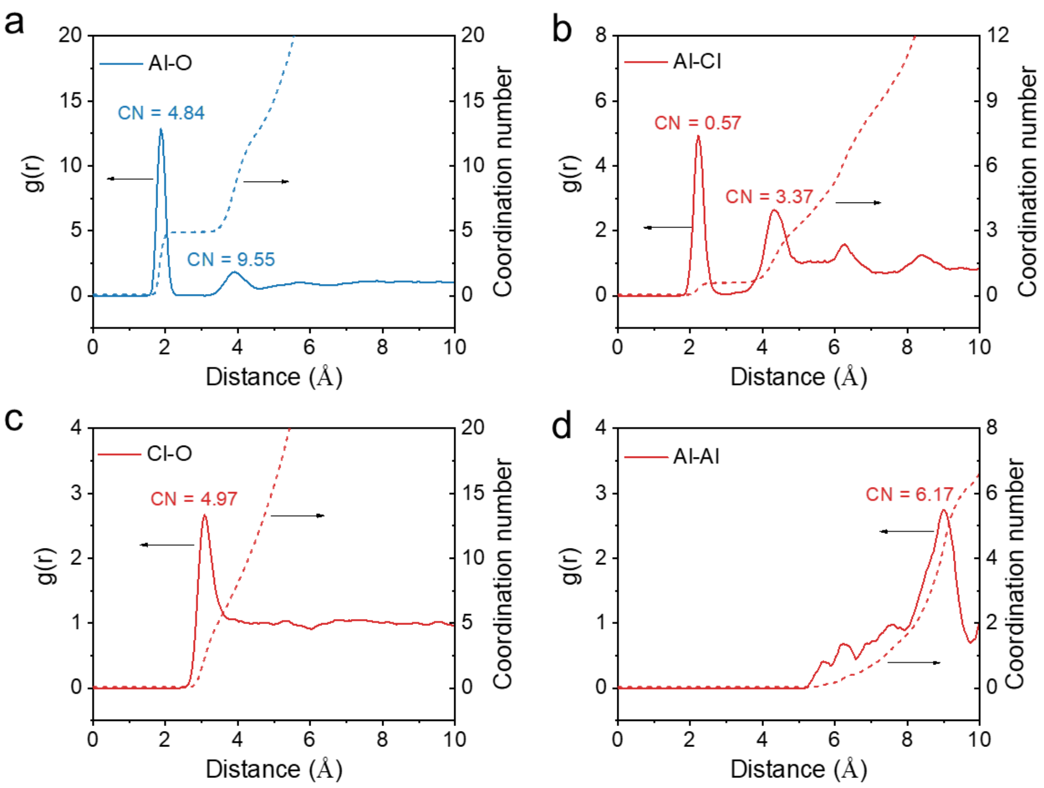

Supplementary Fig. 14 RDFs (solid lines) and integral curves (dashed lines) extracted from AIMD simulations of 3.25 m $AlCl_3$, including **a** Al–O_{water}, **b** Al–Cl, **c** Cl–O_{water}, and **d** Al–Al. Source data are provided as a Source Data file.

Two prominent Al-O_{water} peaks are identified at 1.88 Å and 3.93 Å, indicative of the two solvation sheaths of Al^{3+} with the coordination number (CN) of 4.84 and 9.55 (**Supplementary Fig. 14a**). The majority of Cl (64.1%) are found to occupy the outer solvation shell of Al^{3+} with an Al–Cl distance of 4.33 Å (**Supplementary Fig. 14b**). A small fraction of Cl (17.9% estimated from the CN of 0.57) is located in the inner solvation shell of Al^{3+} with an Al–Cl distance of 2.23 Å. Additionally, Cl exhibits a single distinct solvation shell comprising O atoms from water at a distance of 3.08 Å with the CN of 4.97 (**Supplementary Fig. 14c**). An Al-Al peak is observed at 8.98 Å with the CN of 6.17, indicating the presence of a long-range ordered structure within this electrolyte (**Supplementary Fig. 14d**). The RDF data generated in this study are provided in Supplementary Data 1.

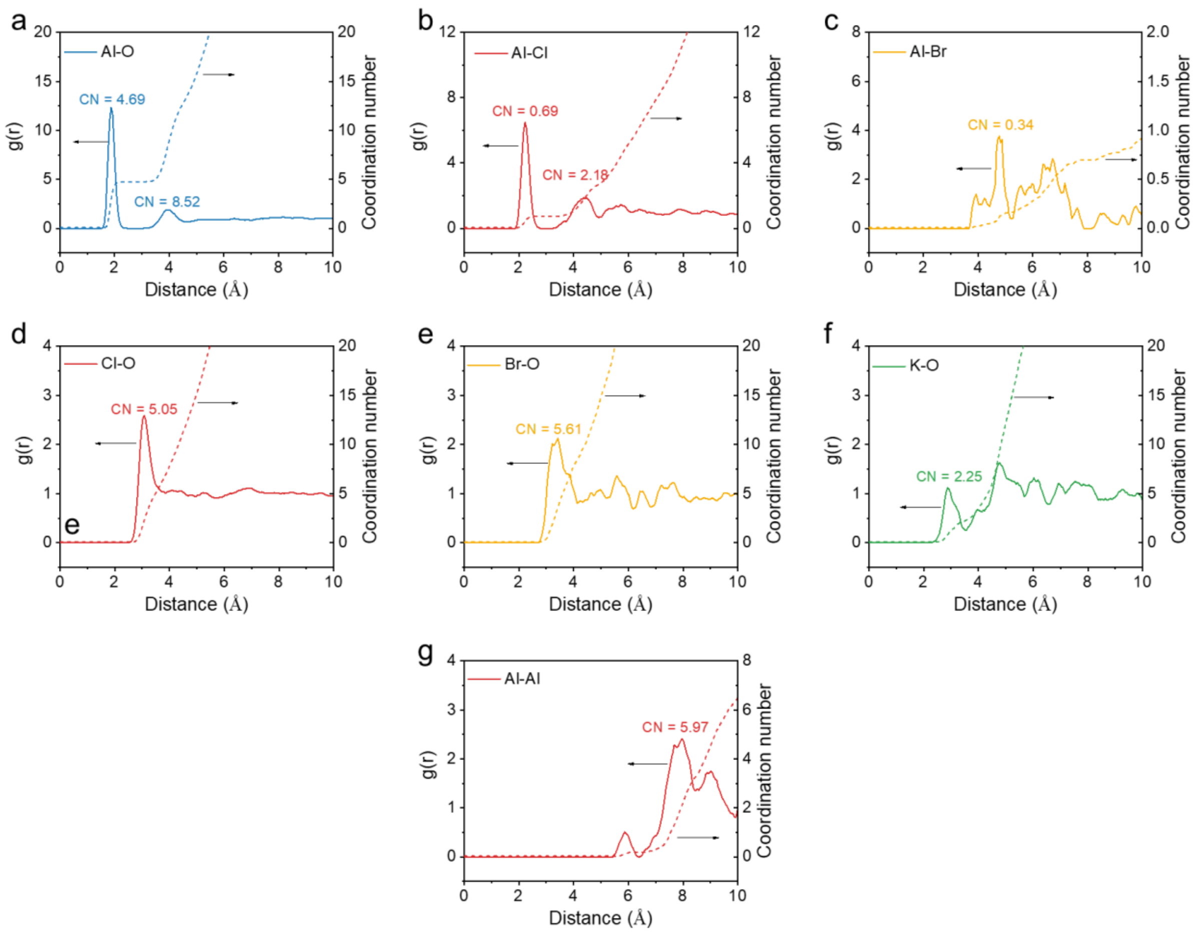

Supplementary Fig. 15 RDFs (solid lines) and integral curves (dashed lines) extracted from AIMD simulations of 3.25 m $AlCl_3$ + 0.5 m KBr including **a** Al–O_{water}, **b** Al–Cl, **c** Al–Br, **d** Cl–O_{water}, **e** Br–O_{water}, **f** K–O_{water}, and **g** Al–Al. Source data are provided as a Source Data file.

Two prominent Al-O_{water} peaks are identified at 1.88 Å and 3.98 Å, indicative of the two solvation sheaths of Al^{3+} with the coordination number (CN) of 4.69 and 8.52 (**Supplementary Fig. 15a**). A significant fraction of Cl (61.5%) resides in the outer solvation sheath of Al^{3+} with an Al–Cl distance of 4.48 Å (**Supplementary Fig. 15b**). A small fraction of Cl (15.4% estimated from the CN of 0.69) is located in the inner solvation sheath of Al^{3+} with an Al–Cl distance of 2.23 Å. Br only resides in the outer solvation of Al^{3+} with an Al–Br distance of 4.48 Å (**Supplementary Fig. 15c**). Cl, Br, and K each exhibit distinct solvation shells composed of O atoms from water: Cl-O_{water} at 3.08 Å with the CN of 5.05 (**Supplementary Fig. 15d**), Br-O_{water} at 3.43 Å with the CN of 5.61 (**Supplementary Fig. 15e**), and K-O_{water} at 2.88 Å with the CN of 2.25 (**Supplementary Fig. 15f**). These solvation interactions contribute to further suppressing water activity. Finally, an Al-Al peak is observed at 7.98 Å with the CN of 5.97, indicating the existence of a long-range ordered structure within this electrolyte (**Supplementary Fig. 15g**). The RDF data generated in this study are provided in Supplementary Data 2.

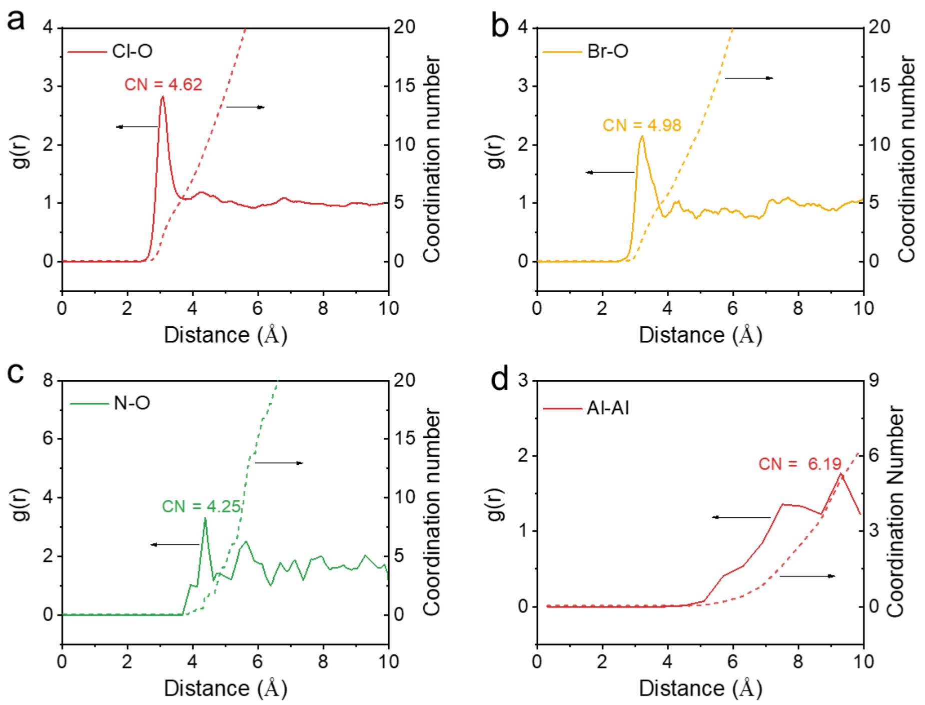

Supplementary Fig. 16 RDFs (solid lines) and integral curves (dashed lines) extracted from AIMD simulations of 3.25 m $AlCl_3$ + 1 m PY14Br including **a** Cl–O_{water}, **b** Br–O_{water}, **c** N–O_{water}, and **d** Al–Al. Source data are provided as a Source Data file.

Cl, Br, and N (from $PY14^+$) each exhibit a distinct solvation shell composed of O atoms from water (**Supplementary Fig. 16a-c**). These solvation shells are identified at 3.08 Å with the CN of 4.62 for Cl-O_{water}, at 3.23 Å with the CN of 4.98 for Br-O_{water}, and at 4.38 Å with the CN of 4.25 for N-O_{water}. These interactions effectively suppress water activity. Additionally, an Al-Al peak is observed at 8.70 Å with the CN of 6.19, indicating the presence of a long-range ordered structure in this electrolyte (**Supplementary Fig. 16d**). The RDF data generated in this study are provided in Supplementary Data 3.

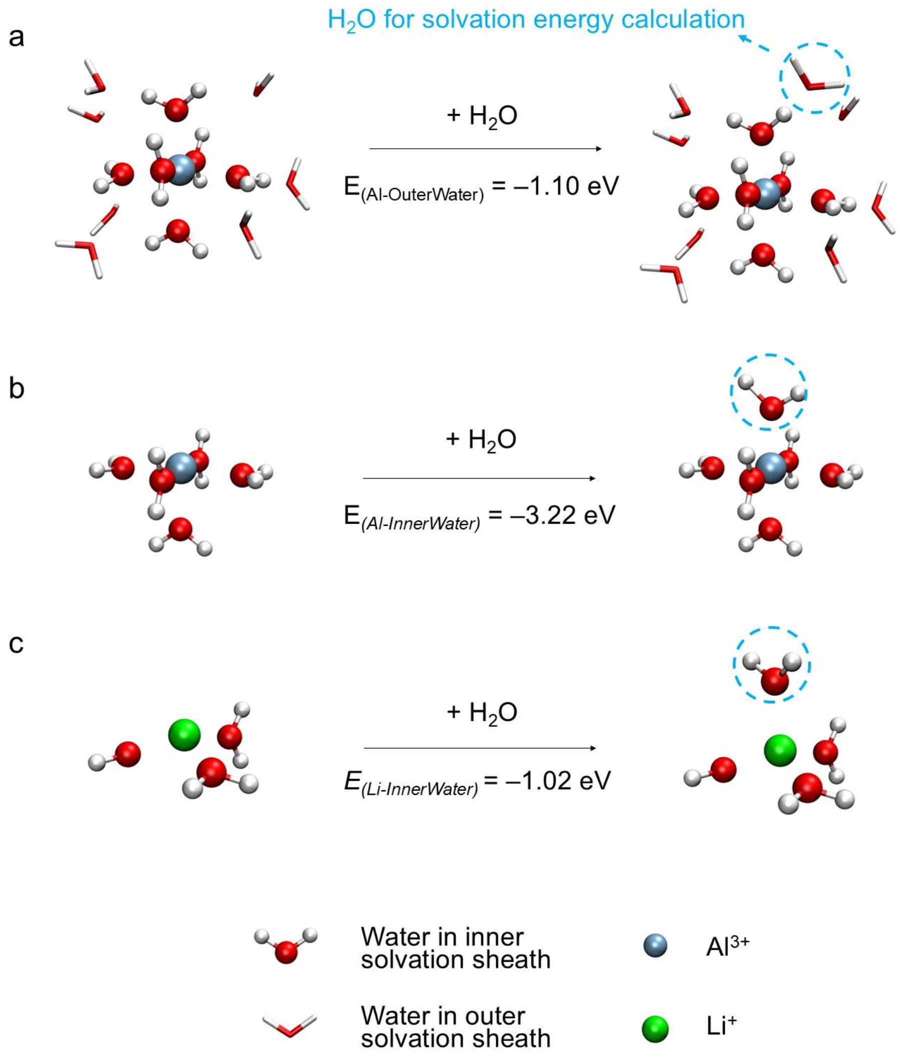

Supplementary Fig. 17 DFT-calculated water solvation energy of **a** outer solvation sheath of Al^{3+}, **b** inner solvation sheath of Al^{3+}, and **c** inner solvation sheath of Li^{+}.

The water solvation energies for the outer solvation sheath of Al^{3+} ($E_{Al\text{-}OuterWater}$), the inner solvation sheath of Al^{3+} ($E_{Al\text{-}InnnerWater}$), and the inner solvation sheath for Li^{+} ($E_{Li\text{-}InnnerWater}$) were calculated using the Vienna Ab initio Simulation Package (VASP) with the PBE exchange-correlation functional. For $E_{Al\text{-}OuterWater}$, we calculated the energy of Al^{3+} with six inner-sheath water molecules and eight outer-sheath water molecules ($E_{Al(InnerWater)_6(OuterWater)_8{}^{3+}}$), the energy of Al^{3+} with six inner-sheath water molecules and seven outer-sheath water molecules ($E_{Al(InnerWater)_6(OuterWater)_7{}^{3+}}$), and the energy of a free water molecule ($E_{FreeWater}$), as illustrated in **Supplementary Fig. 17a**. Using **Supplementary equation (1)**, $E_{Al\text{-}OuterWater}$ was calculated to be –1.10 eV. The atomic coordinates are provided in Supplementary Data 4. For $E_{Al\text{-}InnerWater}$, we calculated the energy of Al^{3+} with six inner-sheath water molecules ($E_{Al(InnerWater)_6{}^{3+}}$), the energy of Al^{3+} with five inner-sheath water molecules ($E_{Al(InnerWater)_5{}^{3+}}$), and the energy of a free water molecule ($E_{FreeWater}$), as illustrated in **Supplementary Fig. 17b**. According to **Supplementary equation (2)**, $E_{Al\text{-}InnerWater}$ was calculated to

be –3.22 eV. The atomic coordinates are provided in Supplementary Data 5. For $E_{Li\text{-}InnerWater}$, we calculated the energy of Li^+ with four inner-sheath water molecules ($E_{Li(InnerWater)_4{}^+}$), the energy of Li^+ with three inner-sheath water molecules ($E_{Li(InnerWater)_3{}^+}$), and the energy of a free water molecule ($E_{FreeWater}$), as illustrated in **Supplementary Fig. 17c**. According to **Supplementary equation (3)**, $E_{Li\text{-}InnerWater}$ was calculated to be –1.02 eV. The atomic coordinates are provided in Supplementary Data 6.

$$E_{Al-OuterWater} = E_{Al(InnerWater)_6(OuterWater)_8{}^{3+}} - E_{Al(InnerWater)_6(OuterWater)_7{}^{3+}} - E_{FreeWater}$$ **(1)**

$$E_{Al-InnerWater} = E_{Al(InnerWater)_6{}^{3+}} - E_{Al(InnerWater)_5{}^{3+}} - E_{FreeWater}$$ **(2)**

$$E_{Li-InnerWater} = E_{Li(InnerWater)_4{}^{+}} - E_{Li(InnerWater)_3{}^{+}} - E_{FreeWater}$$ **(3)**

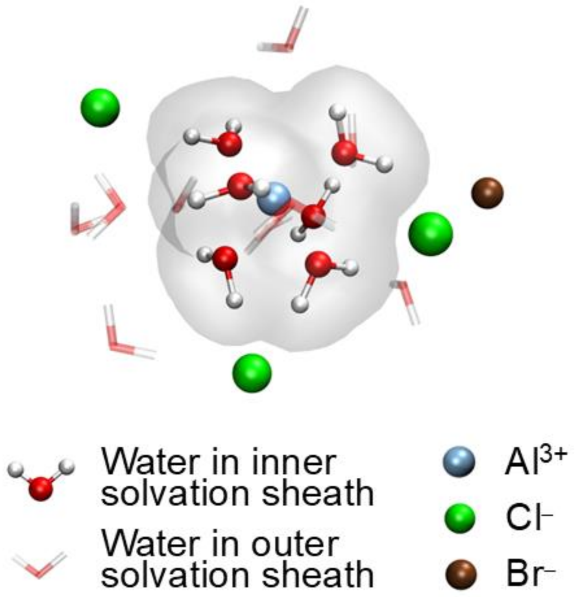

Supplementary Fig. 18 Representative Al^{3+} solvation structure extracted from the snapshot of an AIMD simulated cell for 3.25 m $AlCl_3$ + 1 m PY14Br.

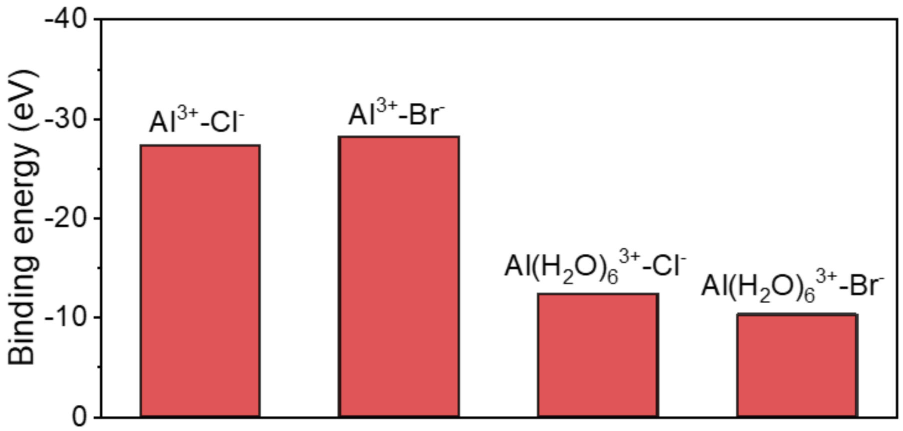

Supplementary Fig. 19 DFT-calculated binding energies of Al^{3+}-X^{-} and $Al(H_2O)_6^{3+}$-X^{-} (X: Cl or Br). Source data are provided as a Source Data file.

The binding energies of Al^{3+}-X^{-} (–27.32 eV for Al^{3+}-Cl^{-} and –28.20 eV for Al^{3+}-Br^{-}) are more than double those of $Al(H_2O)_6^{3+}$-X^{-} (–12.36 eV for $Al(H_2O)_6^{3+}$-Cl^{-} and –10.28 eV for $Al(H_2O)_6^{3+}$-Br^{-}), indicating significantly faster Br^{-}/Cl^{-} dissociation kinetics in $Al(H_2O)_6^{3+}$-X^{-} than in Al^{3+}-X^{-}.

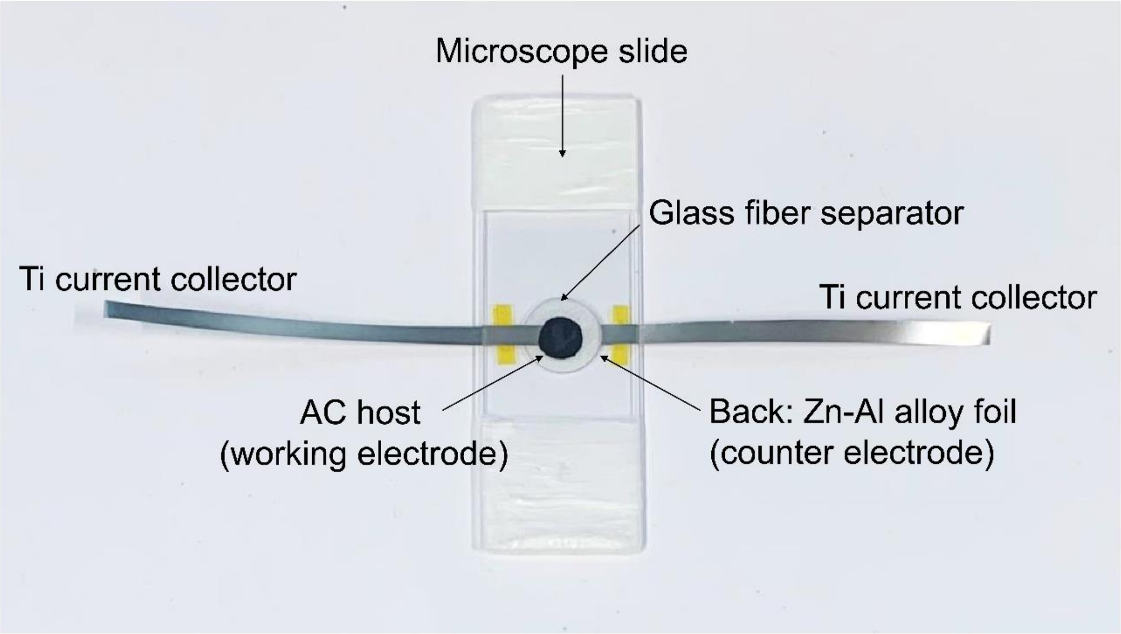

Supplementary Fig. 20 Optical photo of the home-made two-electrode electrochemical device for *in-situ* Raman measurement.

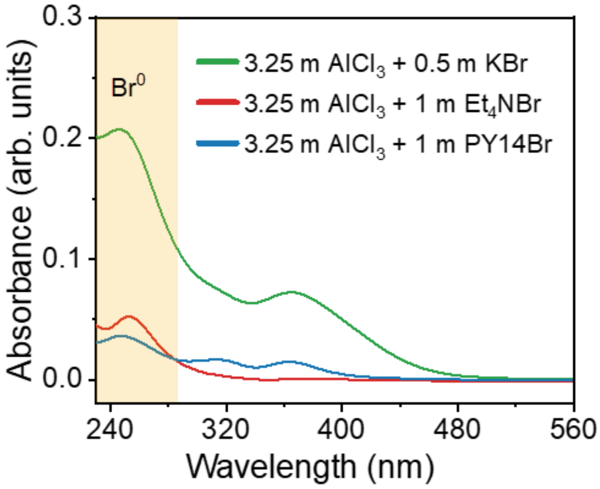

Supplementary Fig. 21 UV-vis absorption spectra of different electrolytes after 10 charge/discharge cycles. Source data are provided as a Source Data file.

After 10 GCD cycles of the AC electrode, 100 μL of the employed electrolyte was extracted from the cell and added to 2.5 mL of the corresponding pristine electrolyte without any electrochemical tests. The resulted solutions, along with the original electrolytes, were subjected to the UV-vis absorption measurement. **Supplementary Fig. 21** presents the signals of the resulted solutions subtracting the signals of the corresponding original electrolytes. The weakened Br^0 peak reflects the reduced dissolution of polybromide species into the electrolyte[1], further supporting the effect of both Et_4N^+ and $PY14^+$ in stabilizing polybromide species. During the $Br^-/Br^0/Br^+$ conversion, Br^0 species are dominantly confined within the electrode rather than being abundantly present in the electrolyte.

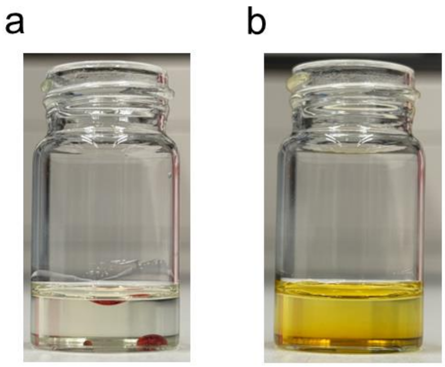

Supplementary Fig. 22 Digital photos of **a** 3.25 m $AlCl_3$ + 1 m PY14Br and **b** 3.25 m $AlCl_3$ + 0.5 m KBr after the galvanostatic charge tests at 1 mA cm^{-2} and 5 mAh cm^{-2} using a three-electrode setup with a glass beaker.

The setup comprised a Ti foil as the working electrode, an over-capacity AC electrode as the counter electrode, and an Ag/AgCl reference electrode. After charging, a water-insoluble phase formed in 3.25 m $AlCl_3$ + 1 m PY14Br, confirming the effect of $PY14^+$ (**Supplementary Fig. 22a**). In contrast, the generated anionic polyhalide species dissolved in 3.25 m $AlCl_3$ + 0.5 m KBr, causing the electrolyte to turn yellow (**Supplementary Fig. 22b**).

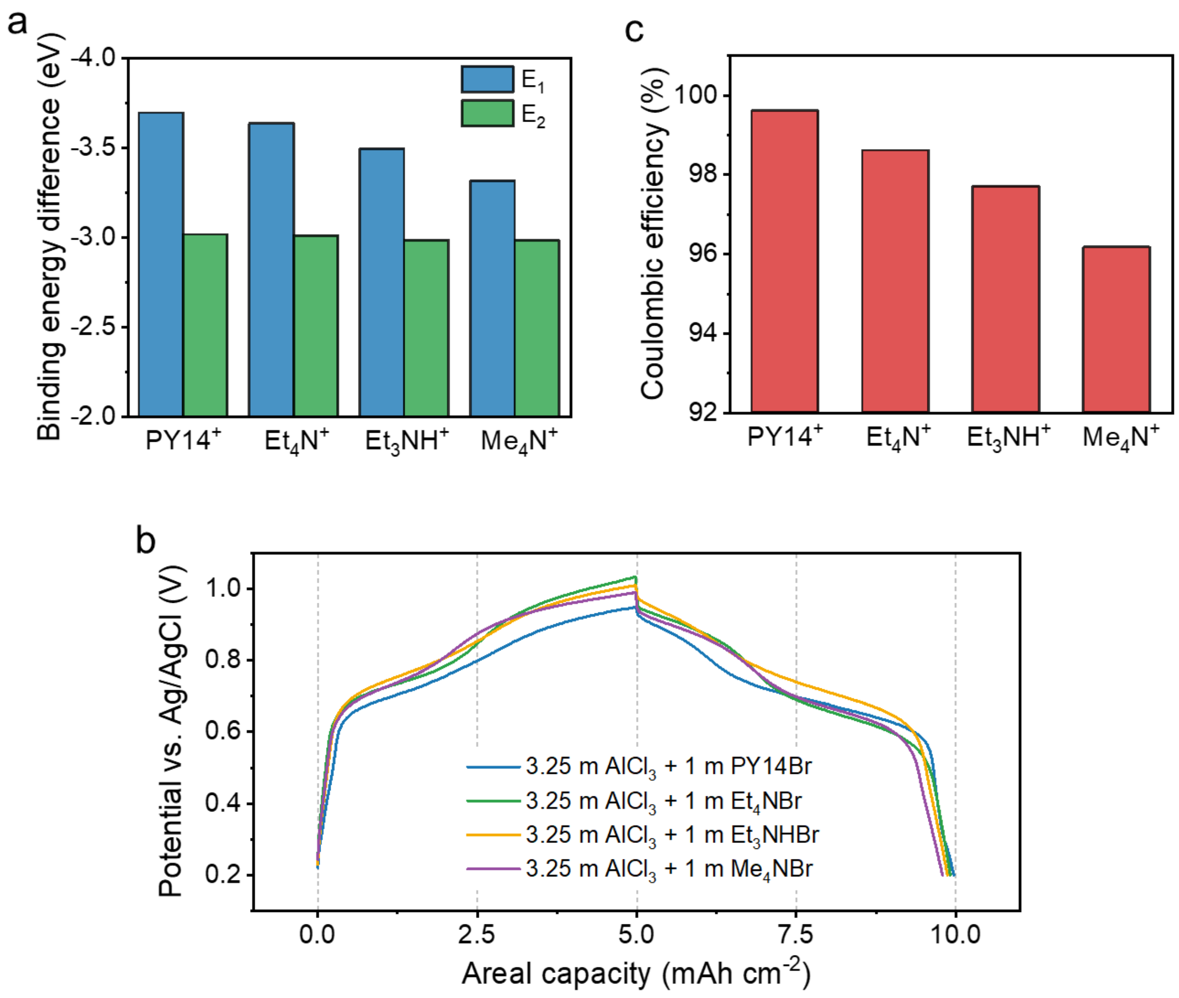

Supplementary Fig. 23 **a** Calculated binding energy differences of various organic cations with Br_3^- and $BrCl_2^-$ relative to with H_2O. **b** GCD profiles of AC electrodes in different electrolytes at 5 mA cm^{-2} and 5 mAh cm^{-2}. **c** Coulombic efficiencies of the AC electrodes using different electrolytes in the three-electrode test. Source data are provided as a Source Data file.

An organic cation with a stronger binding affinity toward anionic polyhalide species than toward water molecules is more likely to promote the formation of stable, water-insoluble phases rather than facilitating their dissolution. To derive data-driven insights, we examined four available bromide salts with bulky organic cations: PY14Br, Et_4NBr, triethylamine hydrobromide (Et_3NHBr), and tetramethylammonium bromide (Me_4NBr). We calculated the binding energies of their cations with Br_3^-, $BrCl_2^-$, and H_2O (**Supplementary Table 5**). All organic cations exhibited stronger bindings with Br_3^-/$BrCl_2^-$ than with H_2O, indicating their tendency to form water-insoluble phases, consistent with experimental observations. To quantify this trend, we defined two parameters: the binding energy difference of the organic cation between with Br_3^- and with H_2O (E_1), the binding energy difference of the organic cation between with $BrCl_2^-$ and with H_2O (E_2). These parameters reflect the relative affinity of the cations toward polyhalide species compared toward water. As shown in

Supplementary Fig. 23a, both E_1 and E_2 follow the order: $PY14^+$ > Et_4N^+ > Et_3NH^+ > Me_4N^+, with the trend in E_1 being more pronounced. This trend is further corroborated by the GCD measurements (**Supplementary Fig. 23b**), where the Coulombic efficiencies of the AC electrodes in the three-electrode test follow the same order (**Supplementary Fig. 23c**): 3.25 m $AlCl_3$ + 1 m PY14Br (99.6%) > 3.25 m $AlCl_3$ + 1 m Et_4NBr (98.6%) > 3.25 m $AlCl_3$ + 1 m Et_3NHBr (97.7%) > 3.25 m $AlCl_3$ + 1 m Me_4NBr (96.2%). These findings highlight E_1 and E_2 as effective descriptors for selecting suitable bromide salts.

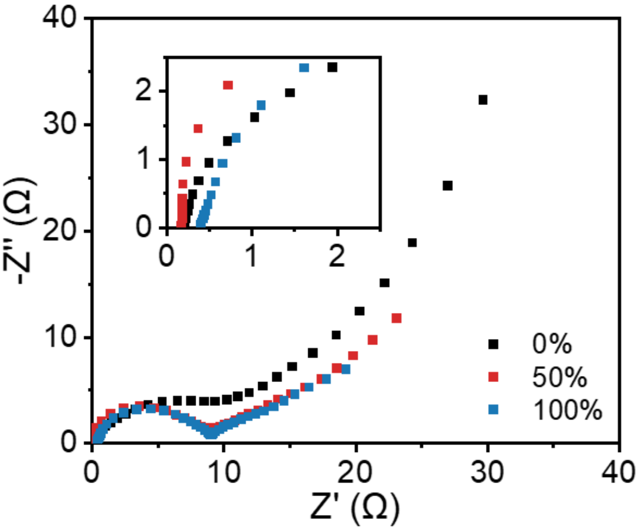

Supplementary Fig. 24 Nyquist plots of the AC electrode at different charge states (0%, 50%, and 100%). The AC electrode maintains a low ohmic resistance (below 0.5 Ω) and charge transfer resistance (below 10 Ω) across all charge states. This observation indicates the fast kinetics of the Br conversion within the AC electrode. Source data are provided as a Source Data file.

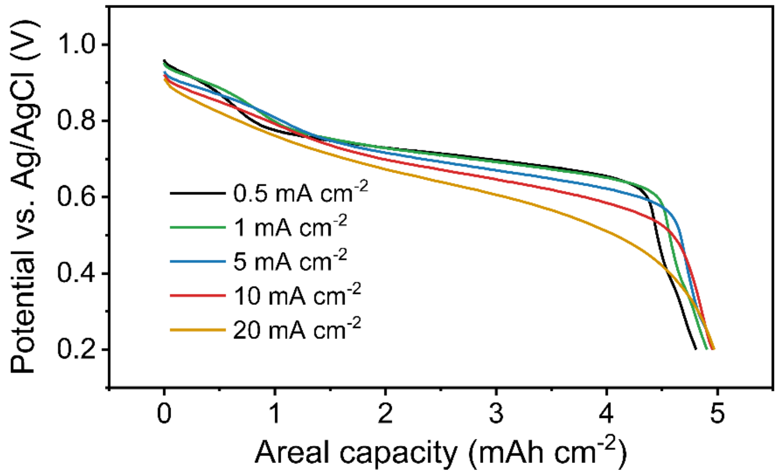

Supplementary Fig. 25 Galvanostatic discharge profiles of the AC electrode at different discharge current densities. The AC electrode was charged at 5 mA cm^{-2} and 5 mAh cm^{-2}. Source data are provided as a Source Data file.

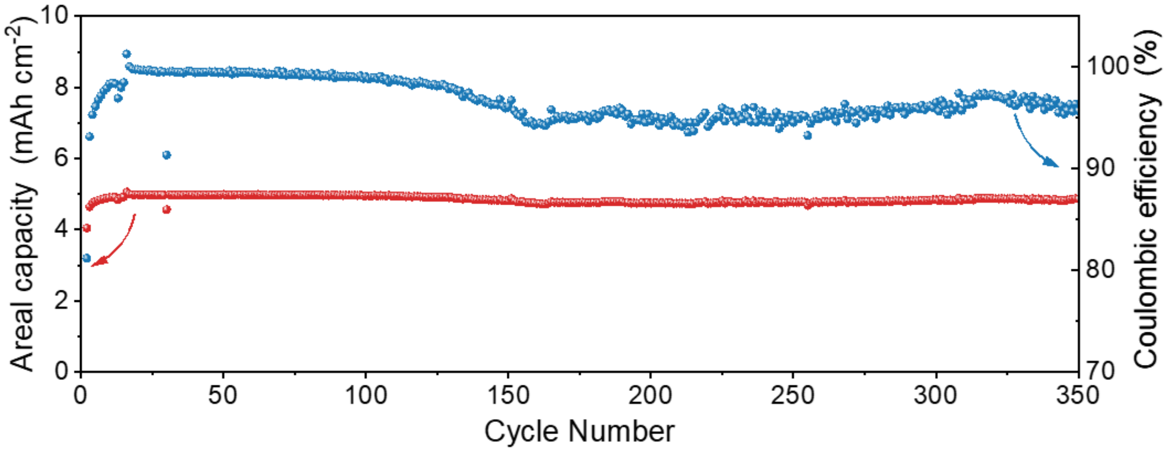

Supplementary Fig. 26 Cycling performance of the AC electrode in 3.25 m $AlCl_3$ + 1 m PY14Br at 1 mA cm^{-2} and 5 mAh cm^{-2}. Source data are provided as a Source Data file.

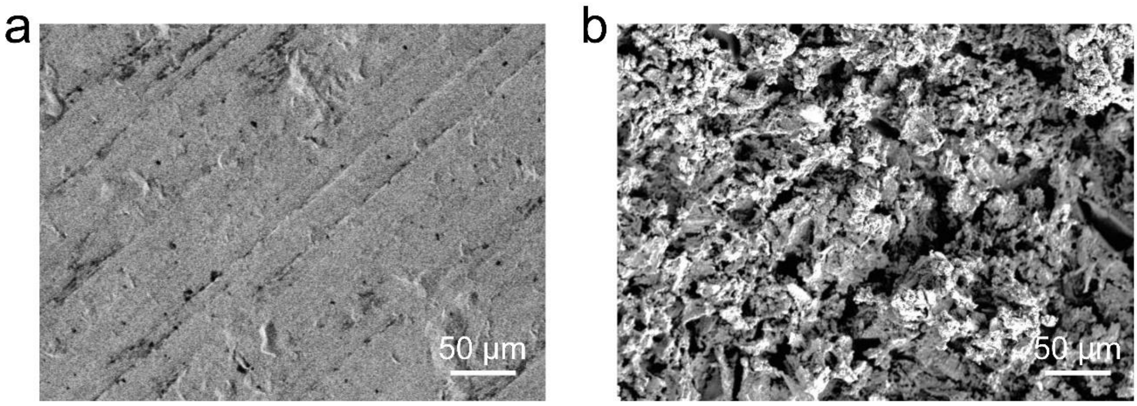

Supplementary Fig. 27 Scanning electron microscope (SEM) images of **a** Zn foil and **b** Zn-Al alloy prepared in 3.25 m $AlCl_3$.

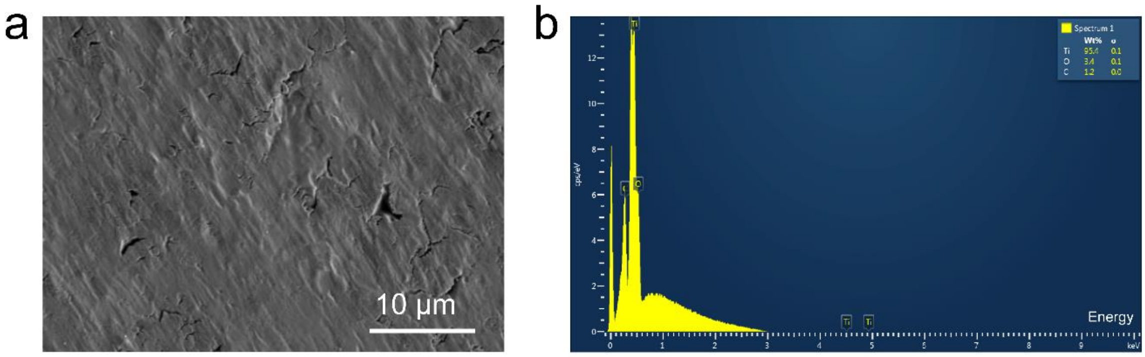

Supplementary Fig. 28 a SEM and **b** EDX spectrum of Ti foil after the LSV test to −1.3 V *vs.* Ag/AgCl in 3.25 m $AlCl_3$ + 1 m PY14Br.

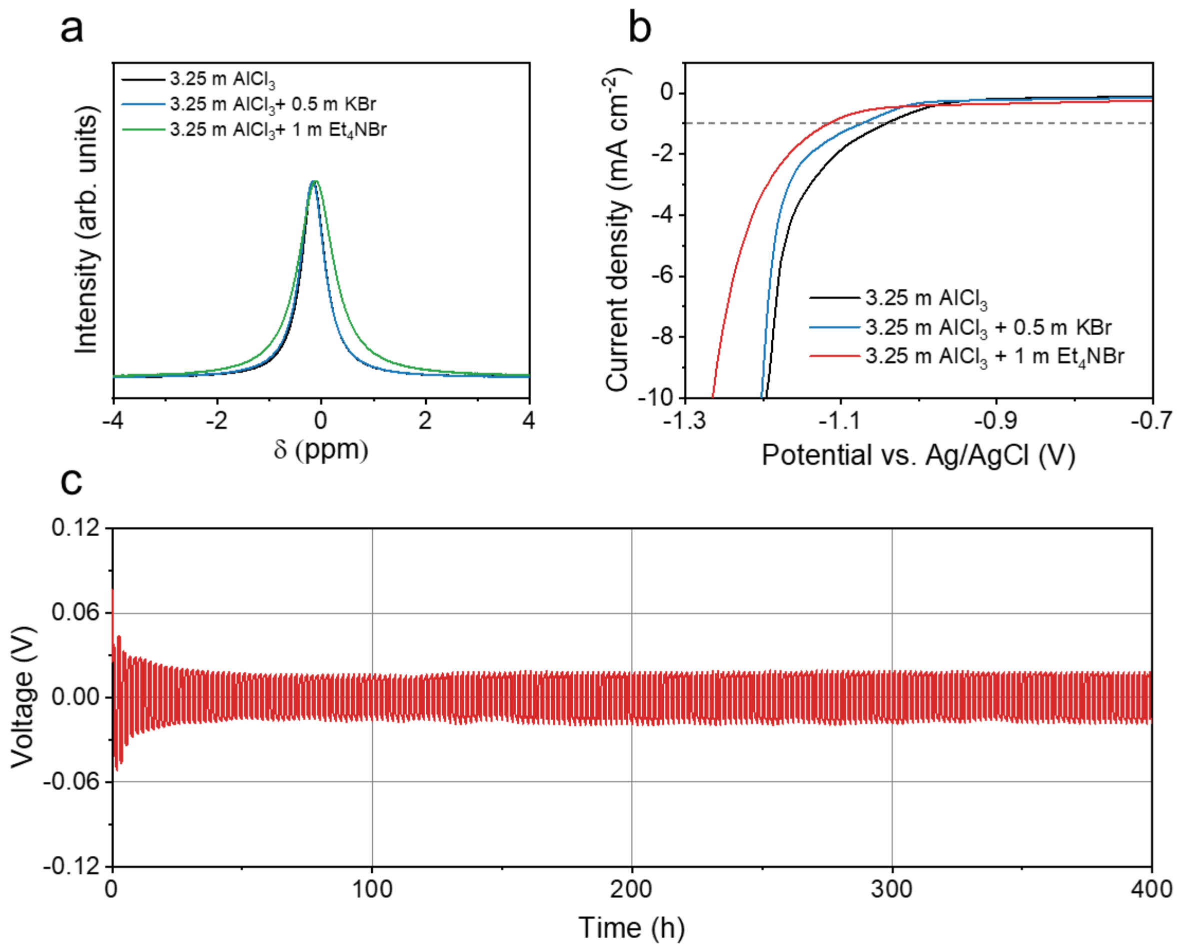

Supplementary Fig. 29 a ^{27}Al NMR spectra of 3.25 m $AlCl_3$, 3.25 m $AlCl_3$ + 0.5 m KBr, and 3.25 m $AlCl_3$ + 1 m Et_4NBr. **b** LSV curves of the Ti electrode at 10 mV s^{-1} in 3.25 m $AlCl_3$, 3.25 m $AlCl_3$ + 0.5 m KBr, and 3.25 m $AlCl_3$ + 1 m Et_4NBr. **c** Galvanostatic stripping/plating of the Zn-Al||Zn-Al symmetric cell in 3.25 m $AlCl_3$ + 1 m Et_4NBr at 1 mA cm^{-2} and 1 mAh cm^{-2}. Source data are provided as a Source Data file.

Compared with 3.25 m $AlCl_3$ + 1 m PY14Br (**Supplementary Fig. 10**, **Fig. 4d**, and **Fig. 4g**), 3.25 m $AlCl_3$ + 1 m Et_4NBr exhibits a similar structure (**Supplementary Fig. 29a)** and performance (**Supplementary Fig. 29b-c**).

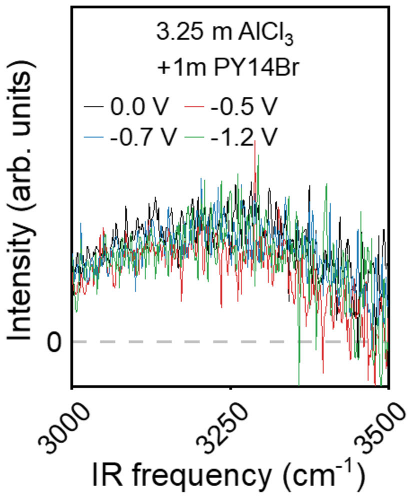

Supplementary Fig. 30 SFG spectra at O–H stretching frequencies of 3.25 m $AlCl_3$ + 1 m PY14Br. Source data are provided as a Source Data file.

The relatively high level of signal noise likely stems from the instability of the $PY14^+$ cations under laser exposure during the SFG measurement. However, the SFG signal change is negligible in this electrolyte at a potential of −1.2 V vs. Ag/AgCl, confirming substantially suppressed hydrogen evolution.

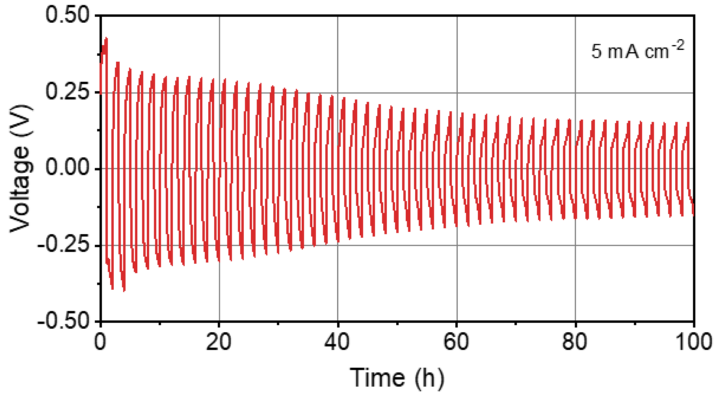

Supplementary Fig. 31 Galvanostatic stripping/plating of the Zn-Al||Zn-Al symmetric cell in 3.25 m $AlCl_3$ + 1 m PY14Br initially at 5 mA cm^{-2} for 100 h as the interfacial activation step. Source data are provided as a Source Data file.

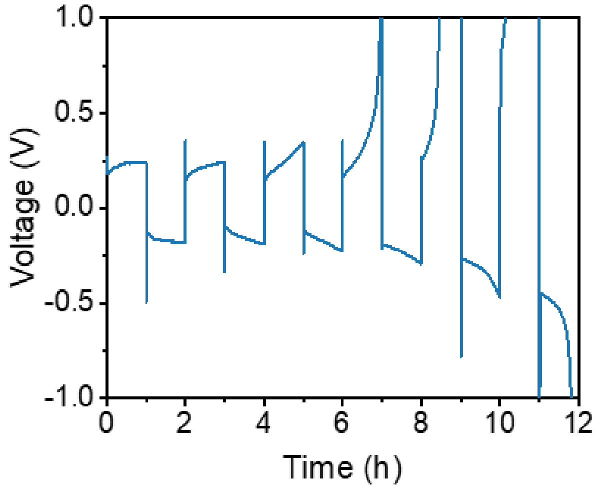

Supplementary Fig. 32 Galvanostatic stripping/plating of the Al||Al symmetric cell in 3.25 m $AlCl_3$ + 1 m PY14Br at 1 mA cm^{-2} and 1 mAh cm^{-2}. Source data are provided as a Source Data file.

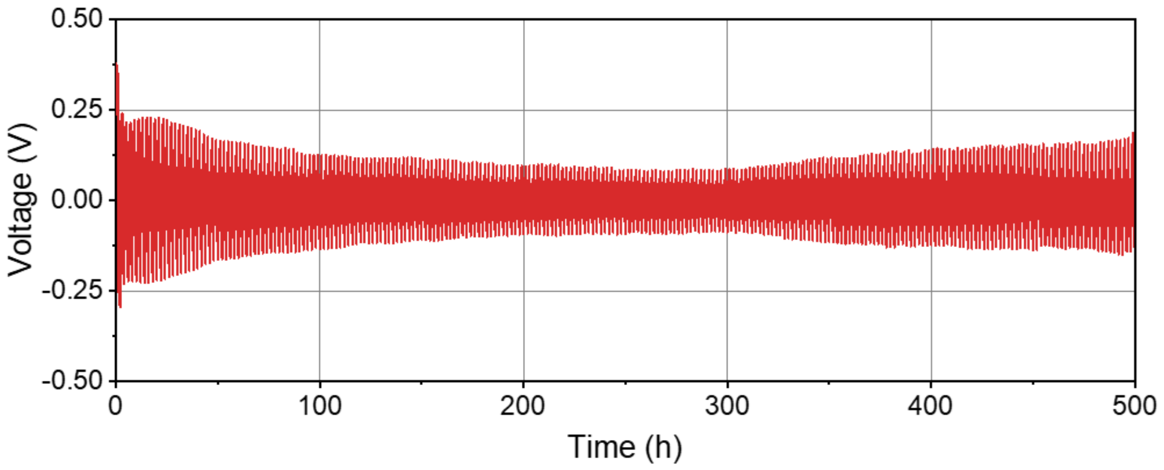

Supplementary Fig. 33 Galvanostatic stripping/plating of the Zn-Al||Zn-Al symmetric cell in 3.25 m $AlCl_3$ + 1 m PY14Br at 5 mA cm^{-2} and 5 mAh cm^{-2}. Source data are provided as a Source Data file.

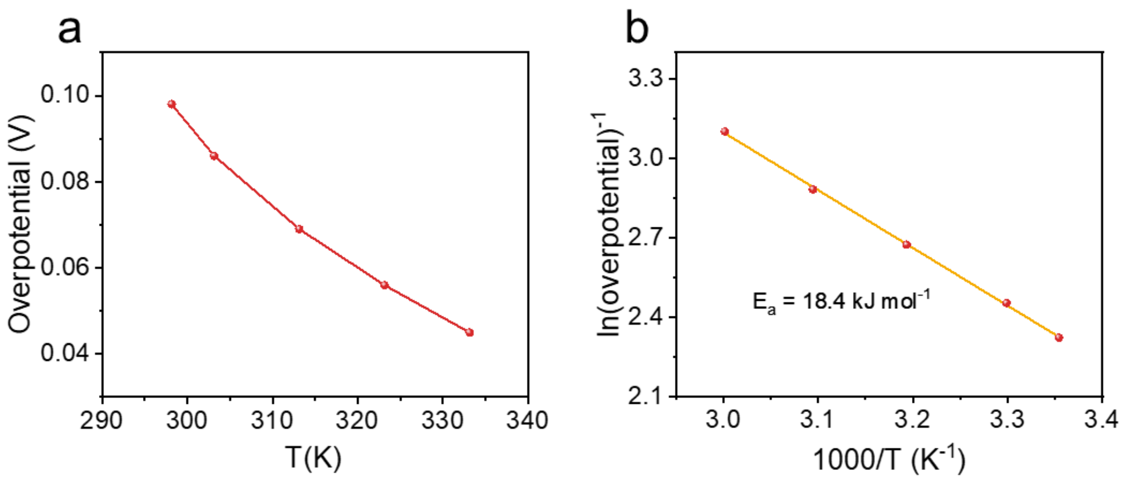

Supplementary Fig. 34 a Temperature dependence of the overpotential for the Zn-Al||Zn-Al symmetric cell in 3.25 m $AlCl_3$ + 1 m PY14Br at 5 mA cm^{-2} and **b** the corresponding Arrhenius plot, revealing an activation energy of 18.4 kJ mol^{-1}. Source data are provided as a Source Data file.

Galvanostatic stripping/plating measurements of the Zn-Al||Zn-Al symmetric cell were performed at 5 mA cm^{-2} and 5 mAh cm^{-2} first for 100 hours at 298.15 K as the activation step. Afterward, the temperature-dependent galvanostatic stripping/plating tests were conducted under the same current density and capacity conditions. The activation energy (E_a) for the cell reaction was determined by fitting the overpotential data using **Supplementary equation (4)**, where T represents the temperature and η represents the overpotential. The calculated E_a value of 18.4 kJ mol^{-2} is comparable to that of other metal stripping/plating reactions, such as ~20 kJ mol^{-2} for Zn anode, and is notably greater than zero. Since metal stripping/plating reaction kinetics positively correlate with temperature ($E_a > 0$), whereas electrical conductivity exhibits the opposite trend ($E_a < 0$)[2], these results collectively confirm the absence of soft short circuiting.

$$E_a = -\frac{T}{1000 \ln(\eta)} \quad \textbf{(4)}$$

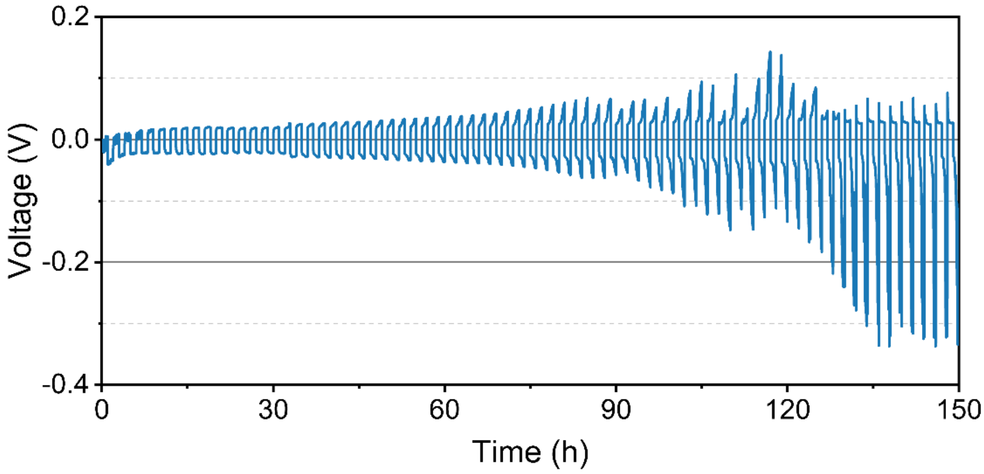

Supplementary Fig. 35 Galvanostatic stripping/plating of the Zn-Al||Zn-Al symmetric cell in 3.25 m $AlCl_3$ at 1 mA cm^{-2} and 1 mAh cm^{-2}. Source data are provided as a Source Data file.

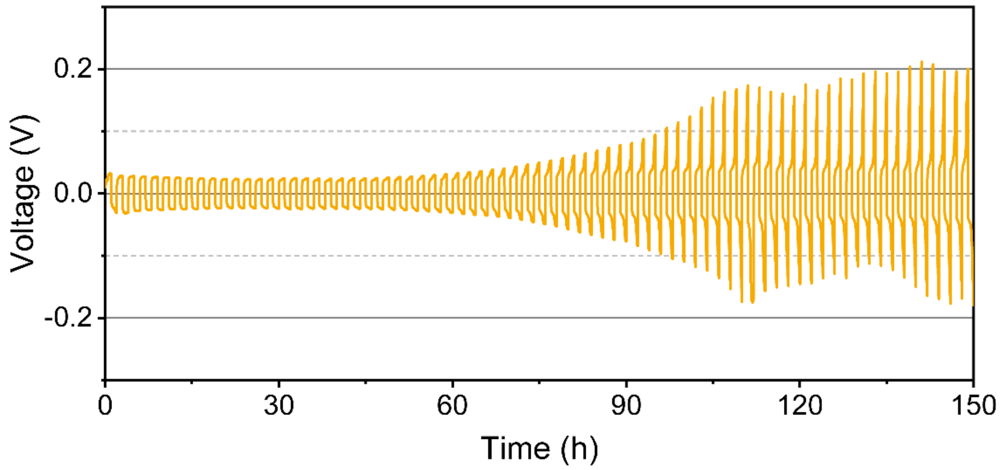

Supplementary Fig. 36 Galvanostatic stripping/plating of the Zn-Al||Zn-Al symmetric cell in 3.25 m $AlCl_3$ + 0.5 m KBr at 1 mA cm^{-2} and 1 mAh cm^{-2}. Source data are provided as a Source Data file.

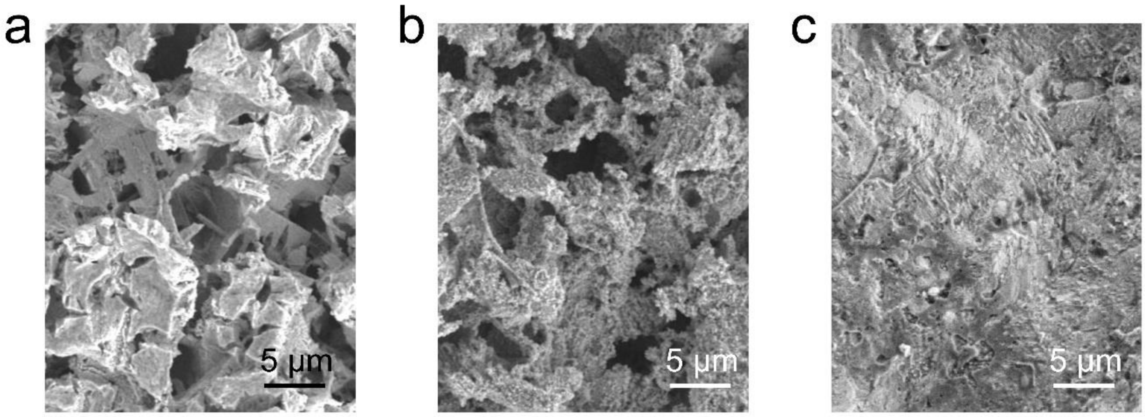

Supplementary Fig. 37 SEM images of Zn-Al alloy after 10 stripping/plating cycles in **a** 3.25 m $AlCl_3$, **b** 3.25 m $AlCl_3$ + 0.5 m KBr, and **c** 3.25 m $AlCl_3$ + 1 m PY14Br.

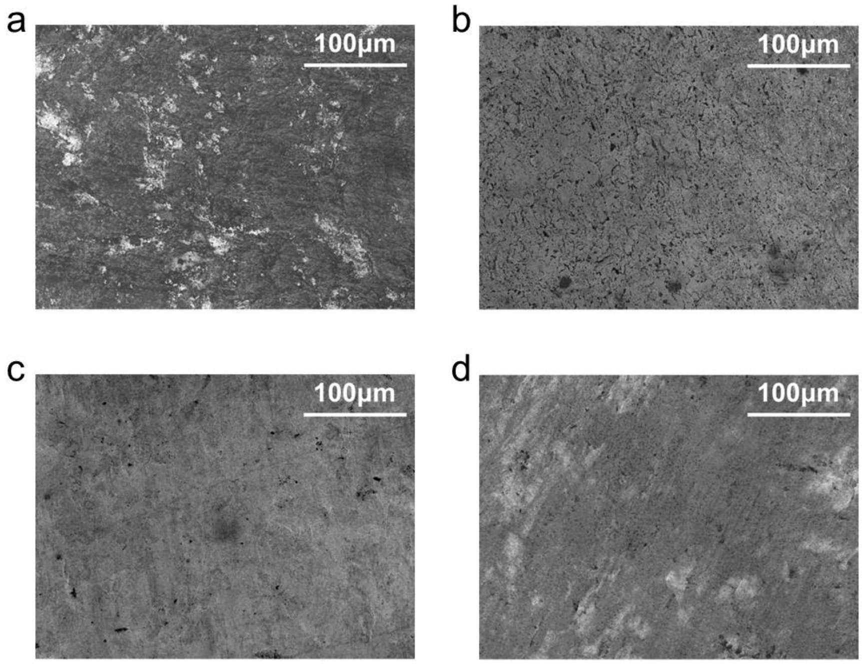

Supplementary Fig. 38 SEM images of **a** the fresh Zn-Al alloy foil, and the Zn-Al alloy foils after immersion in the cycled **b** 3.25 m $AlCl_3$ + 0.5 m KBr, **c** 3.25 m $AlCl_3$ + 1 m PY14Br, and **d** 3.25 m $AlCl_3$ + 1 m Et_4NBr electrolytes.

The cycling tests of the electrolytes were conducted at 5 mA cm^{-2} and 5 mAh cm^{-2} using a three-electrode setup comprising an AC electrode as the hosting electrode, an over-capacity AC electrode as the counter electrode, and an Ag/AgCl reference electrode. After the charge-discharge tests, the cycled electrolytes were extracted from the electrochemical cells, and a fresh Zn-Al alloy foil (**Supplementary Fig. 38a**) was immersed in each electrolyte for 1 hour. Severe corrosion, evidenced by dense surface holes, was observed on the foil immersed in 3.25 m $AlCl_3$ + 0.5 m KBr (**Supplementary Fig. 38b**). In contrast, due to the stabilization mechanism of bulky organic cations that reduces the dissolution of polyhalide into electrolyte and their shuttling to the anode, the corrosion issue was significantly mitigated for the foils immersed in 3.25 m $AlCl_3$ + 1 m PY14Br (**Supplementary Fig. 38c**) and 3.25 m $AlCl_3$ + 1 m Et_4NBr (**Supplementary Fig. 38d**).

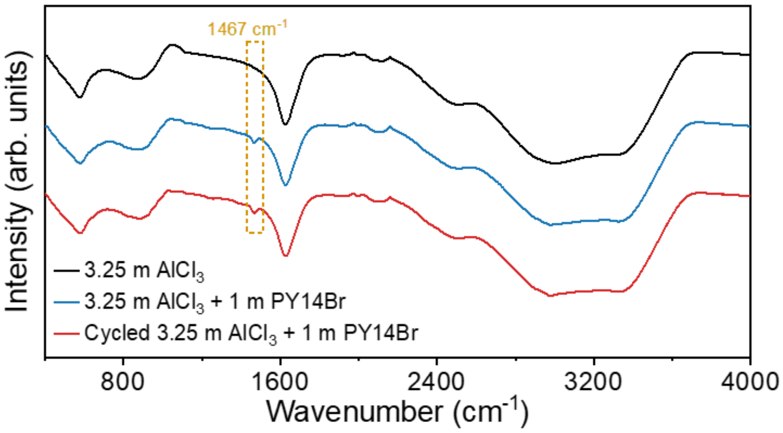

Supplementary Fig. 39 Fourier-transform infrared (FT-IR) spectra of 3.25 m $AlCl_3$, 3.25 m $AlCl_3$ + 1 m PY14Br, and the cycled 3.25 m $AlCl_3$ + 1 m PY14Br electrolyte. Source data are provided as a Source Data file.

We also access the electrochemical stability of PY14Br by analyzing the 3.25 m $AlCl_3$ + 1 m PY14Br electrolyte from an Al-Br cell after 10 charge/discharge cycles at 5 mA cm^{-2} and 5 mAh cm^{-2} via Fourier-transform infrared spectroscopy (FT-IR) and comparing with the original 3.25 m $AlCl_3$ and 3.25 m $AlCl_3$ + 1 m PY14Br electrolytes. As shown in **Supplementary Fig. 39**, the cycled 3.25 m $AlCl_3$ + 1 m PY14Br electrolyte displays a nearly identical FT-IR spectrum to the original one. Notably, the characteristic $PY14^+$ peak at 1467 cm^{-1} remains clearly detectable, confirming the superior electrochemical stability of PY14Br.

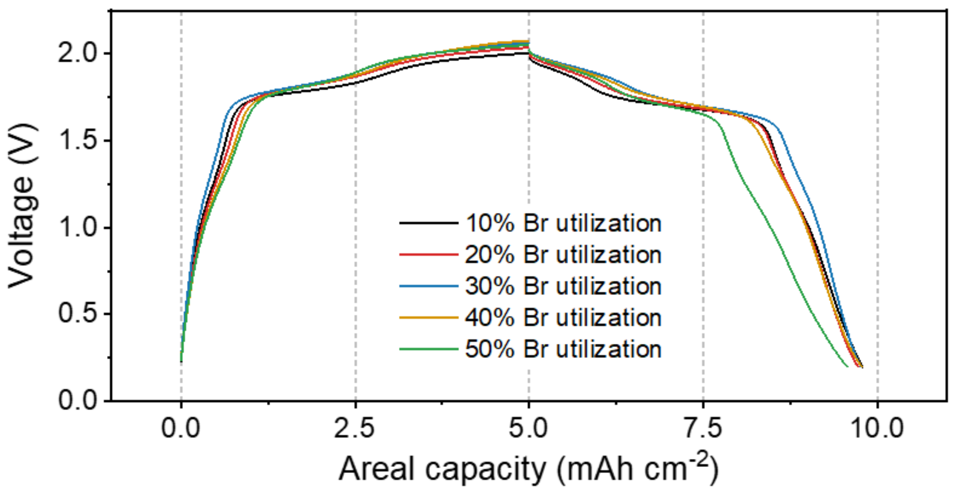

Supplementary Fig. 40 GCD profiles of the Al-Br full cells at different Br utilization efficiencies at 5 mA cm^{-2} with a capacity of 5 mAh cm^{-2} as the charge cutoff. Source data are provided as a Source Data file.

For Br utilization efficiencies ranging from 10% to 40%, the Al-Br cell exhibits nearly identical GCD curves with two distinct charge/discharge plateaus, indicating no significant side effects on the cell performance as the Br utilization efficiency increases within this range. When the Br utilization efficiency reaches 50%, the Al-Br cell shows an obvious performance deterioration.

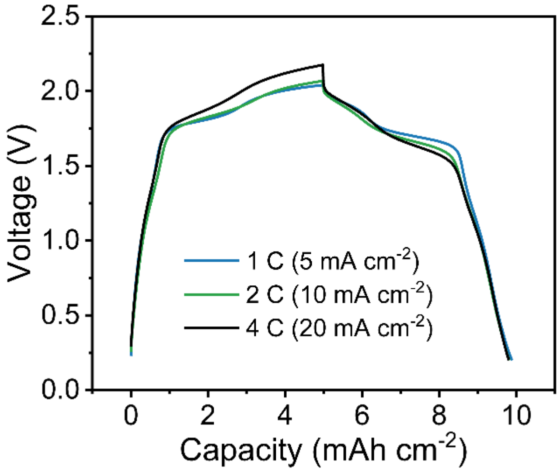

Supplementary Fig. 41 GCD profiles of the aqueous Al-Br full cell at varying current densities. Source data are provided as a Source Data file.

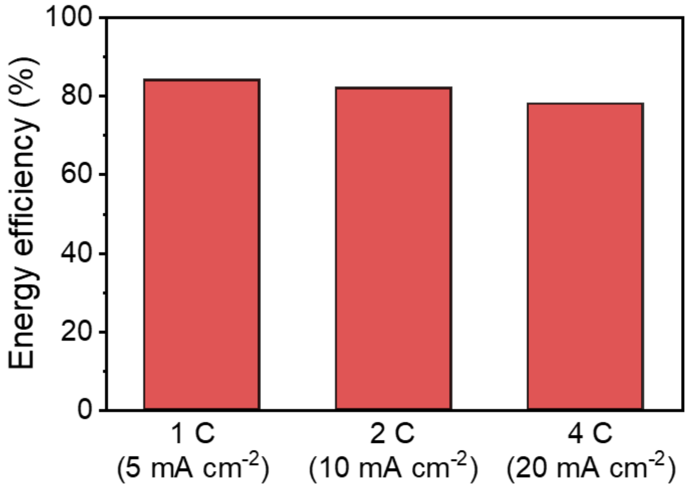

Supplementary Fig. 42 Energy efficiencies of the Al-Br full cell at different current densities. At a current density of 5 mA cm^{-2} (1 C), the full cell achieves a high energy efficiency of 84%. Even at 20 mA cm^{-2} (4 C), the energy efficiency remains as high as 78%, underscoring the high rate capability and practical viability of the Al-Br cell. Source data are provided as a Source Data file.

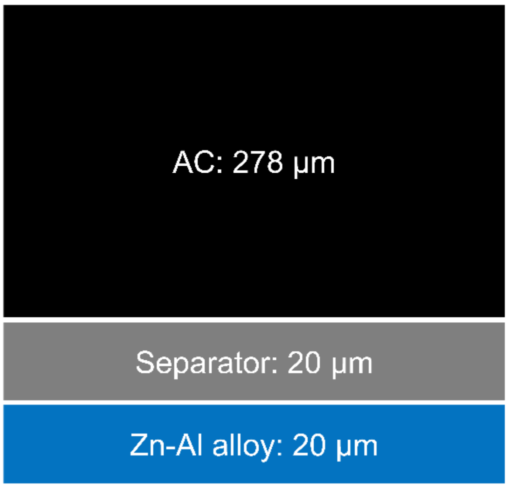

Supplementary Fig. 43 Schematic illustration showing the components of the aqueous Al-Br full cell**.**

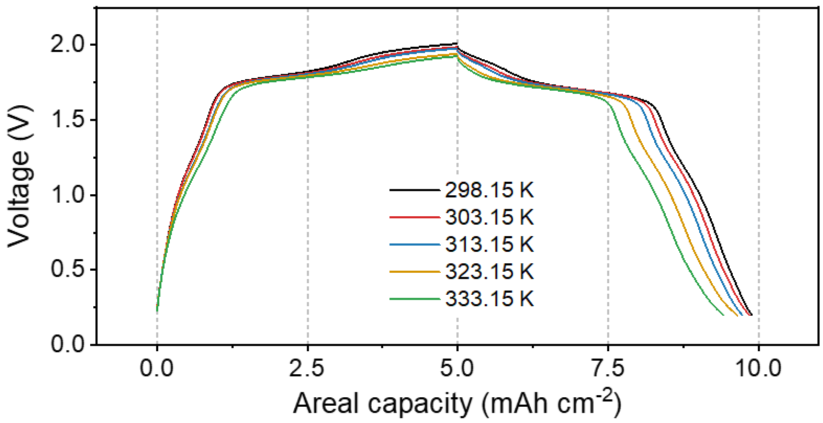

Supplementary Fig. 44 GCD profiles of the Al-Br full cells at different temperature at 5 mA cm^{-2} with a capacity of 5 mAh cm^{-2} as the charge cutoff. Source data are provided as a Source Data file.

Along the temperature increases, the discharge capacity of the Al-Br cell gradually decreases (4.88 mAh cm^{-2} at 298.15 K, 4.85 mAh cm^{-2} at 303.15 K, 4.73 mAh cm^{-2} at 313.15 K, 4.65 mAh cm^{-2} at 323.15 K, and 4.42 mAh cm^{-2} at 333.15 K). This capacity decay can be attributed to the accelerated dissolution of active Br species at elevated temperatures.

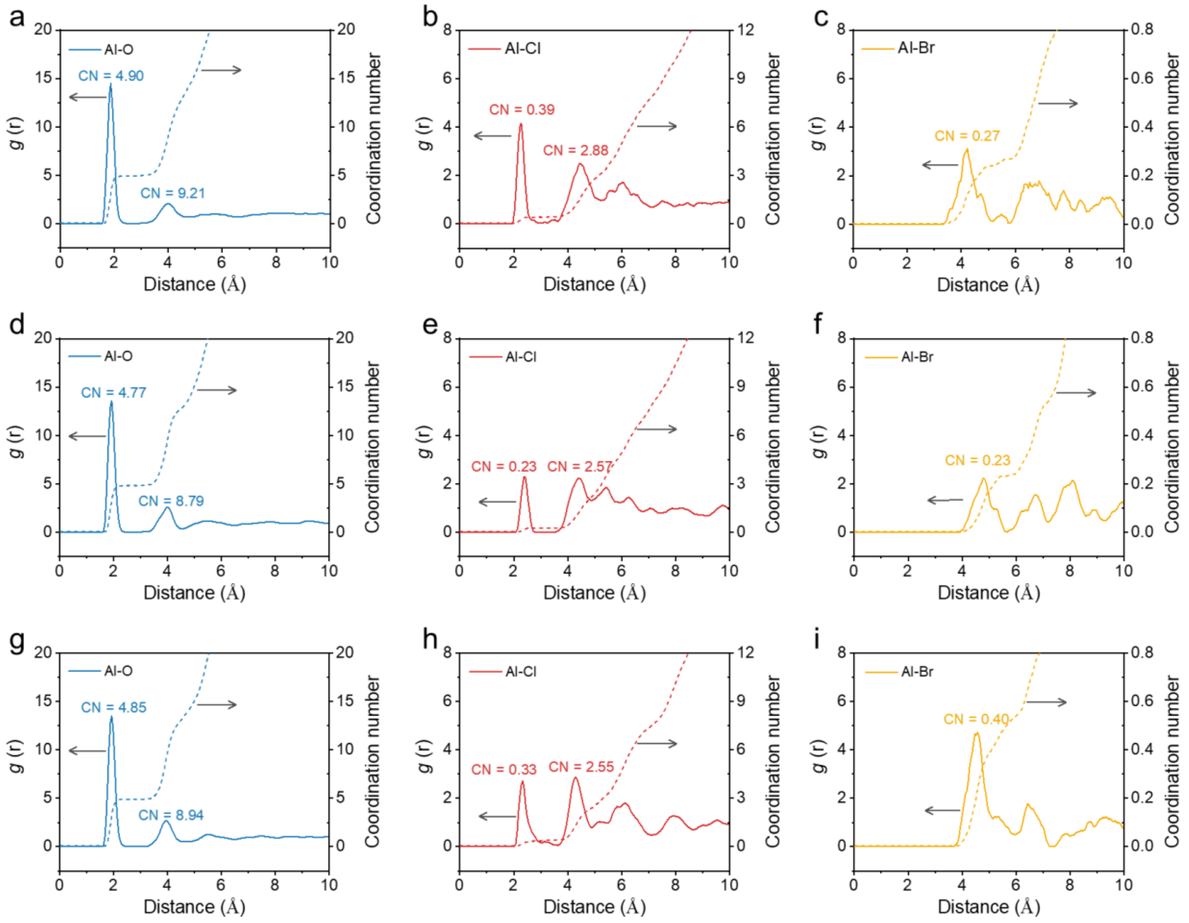

Supplementary Fig. 45 RDFs (solid lines) and integral curves (dashed lines) of Al atoms extracted from AIMD simulations of 3.25 m $AlCl_3$ + 1 m PY14Br at different temperatures, **a** Al–O_{water}, **b** Al–Cl, and **c** Al–Br at room temperature (297.15 K), **d** Al–O_{water}, **e** Al–Cl, and **f** Al–Br at 313.15 K, as well as **g** Al–O_{water}, **h** Al–Cl, and **i** Al–Br at 333.15 K. Source data are provided as a Source Data file.

As reflected by the derived RDFs of Al atoms, the electrolyte maintains a stable structural configuration at elevated temperatures, comparable to that at 298.15 K. Specifically, the majority of water molecules engage in robust ion solvation, while halogen anions reside primarily in the outer solvation sheath of Al^{3+}. These results confirm that the highly structured solvation environment is well preserved over a wide temperature range, consistent with the experimentally observed robust electrochemical performance.

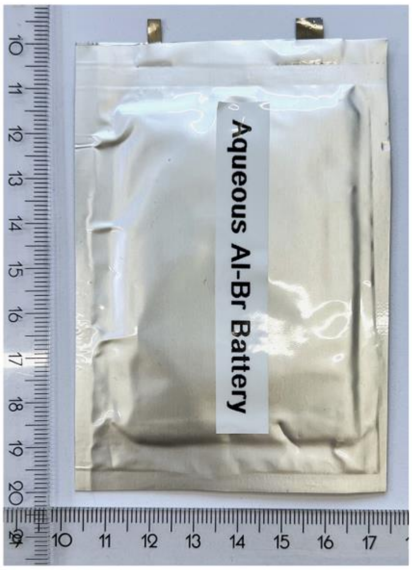

Supplementary Fig. 46 Digital photo of an Al||Br pouch cell.

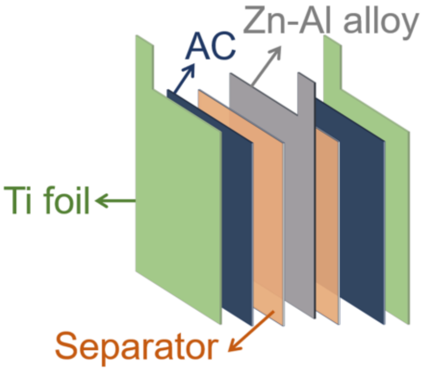

Supplementary Fig. 47 Scheme of the device configuration. Pouch-cell Al-Br devices were assembled by using a two-layer AC cathode (4×6 cm^2, 15 mg cm^{-2}), a one-layer Zn-Al alloy anode (4×6 cm^2, 20 μm in thickness), and GO-coated glass fiber separator (20 μm in thickness). Ti foil was used as the cathode current collector.

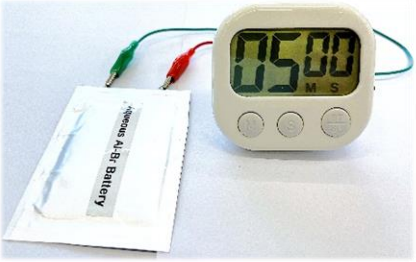

Supplementary Fig. 48 Digital photo of a timer powered by a pouch-cell Al-Br device.

Supplementary Table 1 Viscosity and ionic conductivity of different electrolytes.

**Electrolytes**	**Ionic conductivity (mS cm^{-1})**	**Viscosity (mPa s)**
3.25 m $AlCl_3$	52.9	38
3.25 m $AlCl_3$ + 0.5 m KBr	59.3	42
3.25 m $AlCl_3$ + 1 m PY14Br	32.9	58
3.25 m $AlCl_3$ + 1 m Et_4NBr	36.2	56

The addition of KBr improved the ionic conductivity (59.3 mS cm^{-1} for 3.25 m $AlCl_3$ + 0.5 m KBr) compared with 3.25 m $AlCl_3$ (52.9 mS cm^{-1}) due to the increased ionic concentration. The addition of bulky organic bromide salts reduced the ionic conductivity (32.9 mS cm^{-1} for 3.25 m $AlCl_3$ + 1 m PY14Br and 36.2 mS cm^{-1} for 3.25 m $AlCl_3$ + 1 m Et_4NBr), which can be attributed to the electrolyte viscosity increase (38 mPa s for 3.25 m $AlCl_3$, 58 mPa s for 3.25 m $AlCl_3$ + 1 m PY14Br and 56 mPa s for 3.25 m $AlCl_3$ + 1 m Et_4NBr). It is worth noting that the viscosities and conductivities of these electrolyte are comparable with that of the classic highly concentrated electrolytes, such as 21 m LiTFSI (36 mPa s and 8.2 mS cm^{-1} at 298.15 K) and 1 m LiTFSI + 7 m LiBETI (203 mPa s and 3.0 mS cm^{-1} at 303.15 K)[3,4].

Supplementary Table 2 Cost comparison of key salts used in aqueous Al battery electrolytes.

Salts	Price (€/kg)	Price (€/mol)	Supplier
$AlCl_3 \cdot 6H_2O$	200	48.3	Sigma-Aldrich
PY14Br	4260	946.4	Sigma-Aldrich
Et_4NBr	196	41.2	Sigma-Aldrich
$Al(OTF)_3$	8380	3973.7	Sigma-Aldrich

Supplementary Table 3 Estimated cost of aqueous Al battery electrolytes.

**Electrolytes**	**Price (€/kg)**
3.25 m $AlCl_3$ + 1 m PY14Br	666.7
3.25 m $AlCl_3$ + 1 m Et_4NBr	113.3
2 m $Al(OTF)_3$	4079.8
5 m $Al(OTF)_3$	5895.7

Supplementary Table 4 Analysis of free water molecules in different electrolytes based on the AIMD result.

Electrolyte	Number of free water molecules	Total number of water molecules	Ratio of free water molecules
3.25 m $AlCl_3$	14	225	6.2%
3.25 m $AlCl_3$ + 0.5 m KBr	12	225	5.3%
3.25 m $AlCl_3$ + 1 m PY14Br	4	225	1.8%

Free water molecules were defined as those that are not present in either of the two solvation sheaths of Al^{3+} or in the single solvation sheath of other ions (K^+, $PY14^+$, Cl^-, and Br^-). In this sense, we set cutoff distances between water molecules and the various ions based on the RDF results (**Fig. 2e-g** and **Supplementary Fig. 14-16**, specifically, 5 Å for Al^{3+}, 5 Å for the N atom in $PY14^+$, 4 Å for K^+, 4 Å for Cl^-, and 4 Å for Br^-). Using these criteria, we derived the number of free water molecules in different electrolytes. The ratios of free water molecules in the electrolytes were determined by dividing the number of free water molecules by the total number of water molecules.

Supplementary Table 5 Binding energies of organic cations with Br_3^-, $BrCl_2^-$, and H_2O.

**Organic cation**	**Binding energy with Br_3^- (eV)**	**Binding energy with $BrCl_2^-$ (eV)**	**Binding energy with H_2O (eV)**
$PY14^+$	−4.17423	−3.49277	−0.48032
Et_4N^+	−4.07061	−3.44136	−0.43592
Et_3NH^+	−3.95720	−3.44645	−0.46534
Me_4N^+	−3.85106	−3.51762	−0.53792

Supplementary Table 6 Cathode performance of the reported AABs.

**Cathode materials**	**Mass loading (mg cm^{-2})**	**Areal capacity (mAh cm^{-2})**	**Ref.**
α-MnO_2	2	0.76	[5]
Al_xMnO_2	1	0.478	[6]
Prussian blue analogs	1	0.058	[7]
PANI	1.4	0.259	[8]
S	0.2	0.3	[7]
**AC**	**15**	**4.97**	**This work**

Supplementary Table 7 Elemental quantification of the Zn-Al alloy by inductively coupled plasma optical emission spectrometry (ICP-OES).

Elements	Mass percentage (%)	Atomic percentage (%)
Zn	30.1	14.9
Al	69.9	85.1

Supplementary Table 8 Performance of the aqueous Al-Br cell in comparison with recently reported AZBs, AABs, and NABs.

Battery type	Cathode materials	Electrolyte composition	Mass loading (mg cm^{-2})	Areal capacity (mAh cm^{-2})	Average voltage (V)	Energy density (mWh cm^{-2})	Ref.
AZBs	V_2O_5	1 M $ZnSO_4$	5-7	2.1	0.87	0.18	[9]
AZBs	α-MnO_2	2 M $ZnSO_4$	1-5	1.4	0.61	0.85	[10]
AZBs	Mn_4N	3 M $ZnSO_4$	1.5	0.89	1.4	1.25	[11]
AZBs	Br_2	1.5 M MPIBr + 1.5 M $ZnSO_4$ gel	/	2.2	1.64	3.61	[12]
AZBs	Te	30 m ZnCl2	1.5	1.8	1.1	1.98	[13]
AZBs	PB	1 M $ZnSO_4$	8	0.6	1.33	0.8	[14]
NABs	Graphite	$AlCl_3$/EMIMCl	8.6	0.95	2.0	1.89	[15]
NABs	Graphene	$AlCl_3$/EMIMCl	4	0.38	2.0	0.76	[16]
NABs	Poly-phenazine	$AlCl_3$/EMIMCl	1	0.12	0.8	0.096	[17]
NABs	Amine	$AlCl_3$/EMIMCl	12.9	1.61	1.1	1.78	[18]
NABs	S	$AlCl_3$/EMIMCl	6.5	1.6	0.75	1.2	[19]
NABs	Te	$AlCl_3$/EMIMCl	1	0.91	1.0	0.91	[20]
NABs	Se	NaCl-KCl-$AlCl_3$	3.5	2.27	0.9	2.05	[21]
AABs	α-MnO_2	2 m $Al(CF_3SO_3)_3$	2	0.76	1.3	0.99	[5]
AABs	Al_xMnO_2	2 M $Al(OTF)_3$	1	0.48	1.5	0.72	[6]
AABs	KNHCF	0.5 M $Al_2(SO_4)_3$	1	0.045	1.1	0.05	[22]
AABs	CoHCF	3 M $AlCl_3$	10	1.04	1.56	1.62	[23]
AABs	PANI	$Al(ClO_4)_3 \cdot 9H_2O$/SN	1.4	0.26	0.7	0.18	[8]
AABs	S	0.5 M $Al_2(SO_4)_3$	0.2	0.3	0.5	0.15	[7]
**AABs**	**AC**	**3.25 m AlCl3 + 1 m PY14Br**	**15**	**4.89**	**1.7**	**8.31**	**This work**

Supplementary References

1 Wei, H. *et al.* Boosting aqueous non-flow zinc–bromine batteries with a two-dimensional metal–organic framework host: an adsorption-catalysis approach. *Energy Environ. Sci.* **16**, 4073-4083 (2023).

2 Li, Q., Chen, A., Wang, D., Pei, Z. & Zhi, C. "Soft shorts" hidden in zinc metal anode research. *Joule* **6**, 273-279 (2022).

3 Suo, L. *et al.* “Water-in-salt” electrolyte enables high-voltage aqueous lithium-ion chemistries. *Science* **350**, 938-943 (2015).

4 Yamada, Y. *et al.* Hydrate-melt electrolytes for high-energy-density aqueous batteries. *Nat. Energy* **1**, 16129 (2016).

5 Zhao, Q. *et al.* Solid electrolyte interphases for high-energy aqueous aluminum electrochemical cells. *Sci. Adv.* **4**, eaau8131 (2018).

6 Ran, Q. *et al.* Aluminum-copper alloy anode materials for high-energy aqueous aluminum batteries. *Nat. Commun.* **13**, 576 (2022).

7 Yan, C. *et al.* Reversible Al metal anodes enabled by amorphization for aqueous aluminum batteries. *J. Am. Chem. Soc.* **144**, 11444-11455 (2022).

8 Meng, P. *et al.* A low-cost and air-stable rechargeable aluminum-ion battery. *Adv. Mater.* **34**, 2106511 (2022).

9 Kundu, D., Adams, B. D., Duffort, V., Vajargah, S. H. & Nazar, L. F. A high-capacity and long-life aqueous rechargeable zinc battery using a metal oxide intercalation cathode. *Nat. Energy* **1**, 16119 (2016).

10 Pan, H. *et al.* Reversible aqueous zinc/manganese oxide energy storage from conversion reactions. *Nat. Energy* **1**, 16039 (2016).

11 Liu, Y. *et al.* A new design strategy enables high Mn-utilization rate in aqueous zinc–manganese batteries: Constructing cathodic local Mn-rich region. *Adv. Energy Mater.* **14**, 2304161 (2024).

12 Dai, C. *et al.* Fast constructing polarity-switchable zinc-bromine microbatteries with high areal energy density. *Sci. Adv.* **8**, eabo6688 (2022).

13 Du, J. *et al.* A high-energy tellurium redox-amphoteric conversion cathode chemistry for aqueous zinc batteries. *Adv. Mater.* **36**, 2313621 (2024).

14 Zhang, L., Chen, L., Zhou, X. & Liu, Z. Towards high-voltage aqueous metal-ion batteries beyond 1.5 V: The zinc/zinc hexacyanoferrate system. *Adv. Energy Mater.* **5**, 1400930 (2015).

15 Elia, G. A., Kyeremateng, N. A., Marquardt, K. & Hahn, R. An aluminum/graphite battery with ultra-high rate capability. *Batteries & Supercaps* **2**, 83-90 (2019).

16 Chen, H. *et al.* Ultrafast all-climate aluminum-graphene battery with quarter-million cycle life. *Sci. Adv.* **3**, eaao7233

17 Grieco, R. *et al.* A phenazine-based conjugated microporous polymer as a high performing cathode for aluminium–organic batteries. *Faraday Discuss.* **250**, 110-128 (2024).

18 Wang, G. *et al.* An efficient rechargeable aluminium–amine battery working under quaternization chemistry. *Angew. Chem. Int. Ed.* **61**, e202116194 (2022).

19 Gao, T. *et al.* A rechargeable Al/S battery with an ionic-liquid electrolyte. *Angew. Chem. Int. Ed.* **55**, 9898-9901 (2016).

20 Jiao, H., Tian, D., Li, S., Fu, C. & Jiao, S. A rechargeable Al–Te battery. *ACS Applied Energy Materials* **1**, 4924-4930 (2018).

21 Pang, Q. *et al.* Fast-charging aluminium–chalcogen batteries resistant to dendritic shorting. *Nature* **608**, 704-711 (2022).

22 Jia, B.-E. *et al.* Laminated tin–aluminum anodes to build practical aqueous aluminum batteries. *Energy Storage Mater.* **65**, 103141 (2024).

23 Yuan, X. *et al.* An aqueous rechargeable Al-ion battery based on cobalt hexacyanoferrate and Al metal. *Adv. Energy Mater.* **14**, 2302712 (2024).
