# Supplementary material for: Hydrate-melt electrolyte design for aqueous aluminium-bromine batteries with enhanced energy-power merits
Source: Nat Commun. 2025 Jul 9;16:6329. doi: 10.1038/s41467-025-61740-y (PMC12241333; doi:10.1038/s41467-025-61740-y)
Supplement: Supplementary file 2 — Description of Additional Supplementary Files [file 41467_2025_61740_MOESM2_ESM.pdf]

Description of Additional Supplementary Files

File Name: Supplementary Data 1

Description: Atomic coordinates of $AlCl_3$ after 30 ps AIMD simulations.

File Name: Supplementary Data 2

Description: Atomic coordinates of 3.25 m $AlCl_3$ + 0.5 m KBr after 30 ps AIMD simulations.

File Name: Supplementary Data 3

Description: Atomic coordinates of 3.25 m $AlCl_3$ + 1 m PY14Br after 30 ps AIMD simulations.

File Name: Supplementary Data 4

Description:Atomic coordinates of $Al(InnerWater)_6(OuterWater)_8^{3+}$.

File Name: Supplementary Data 5

Description:Atomic coordinates of $Al(InnerWater)_6^{3+}$.

File Name: Supplementary Data 6

Description:Atomic coordinates of $Li(InnerWater)_4^{+}$.
